# The effects of feeding guild, seasonality, and warming on the gut microbiomes of Antarctic echinoderms

**DOI:** 10.1186/s12866-026-05114-4

**Published:** 2026-05-12

**Authors:** Kudzai Hwengwere, Benjamin H. Gregson, Susannah J. Salter, Emma Bolton, Lama Alqahtani, Sylvia Rofael, Vitor H. Teixeira, Timothy D. McHugh, Grant G. January, Lloyd S. Peck, Mathew Upton, Melody S. Clark

**Affiliations:** 1 British Antarctic Survey, High Cross, Madingley Road, Cambridge, CB3 0ET UK; 2 Marine Biology and Ecology Research Centre, School of Biological and Marine Sciences, University of Plymouth, Drake Circus, Plymouth, PL4 8AA UK; 3 Present Address: MRC, Laboratory of Medical Sciences, Imperial College Hammersmith Campus, Du Cane Road, London, W12 0HS UK; 4 School of Life Sciences, Faculty of Science and Engineering, Anglia Ruskin University, East Road Cambridge, Cambridge, CB1 1PT UK; 5 Department of Veterinary Medicine, University of Cambridge, Cambridge, CB3 0ES UK; 6 Basecamp Research Ltd, Unit 510 Clerkenwell Workshops, 27 Clerkenwell Close, London, EC1R 0AT UK; 7 UCL Respiratory, Division of Medicine, University College London, London, UK; 8 Bioengineering institute, Health Sector, King Abdulaziz City for Science and Technology, Riyadh, 11442 Saudi Arabia; 9 UCL, Centre for Clinical Microbiology, Royal Free Campus, London, NW3 2QG UK; 10 Faculty of Pharmacy, Alexandria University, Alexandria, Egypt; 11 School of Biomedical Sciences, Derriford Research Facility, University of Plymouth, Drake Circus, Plymouth, PL4 8AA UK

**Keywords:** Marine, Invertebrate, Gut microbiome, Metabarcoding, Diet, Season, Holothurian, Sea star, Brittle star, Sea urchin

## Abstract

**Background:**

Antarctic marine food webs are expected to be significantly impacted by future climate change. In particular, the recent rapid regional warming in the West Antarctic Peninsula has, and will continue to have, a negative impact on endemic marine biodiversity. However, despite the growing recognition of the role microbial symbionts play in mediating responses to environmental change, microbiome characterisation has been conducted for only a small fraction of the marine invertebrates in the Southern Ocean. Our study examined the effects of feeding guild, seasonality, and experimental warming (6 months at + 2 °C) on the gut microbiome of six species of near-shore marine Antarctic echinoderms sampled from waters off Rothera Research Station, Antarctica. Our study used 16 S rRNA amplicon sequencing of the V3-V4 region, with analyses including measurements of alpha and beta-diversity alongside co-occurrence network analyses.

**Results:**

Of the six invertebrate species sampled in winter, peak species diversity values in gut microbiomes were observed in the omnivores, *Ophionotus victoriae* and *Sterechinus neumayeri*, with lower values in the scavenger/predator, *Odontaster validus*, and the suspension feeders, *Cucumaria georgiana*,* Echinopsolus charcoti*, and *Heterocucumis steineni*. In the seasonal experiment, *H. steineni* bacterial gut species diversity doubled from winter to early summer yet decreased by a similar magnitude during the same period in *O. victoriae*. Despite these opposing diversity trends, both species displayed similar increases in the relative abundances of *Bacteroidota* and *Bacillota* in winter and early summer in their gut microbiomes. Bacterial diversity in the gut microbiome of the sea cucumbers *E. charcoti* and *H. steineni*, was not impacted by six-months at + 2 ˚C above ambient, although *C. georgiana* displayed a decrease in observed ASVs following this treatment.

**Conclusions:**

These results suggest a strong influence of feeding guild and seasonality on the gut microbiomes of these invertebrates. There appeared to be little effect of warming (+ 2 °C) on the taxonomic composition of the gut microbiomes of the three holothurians. This highlights the need to examine the functional significance of experimental warming treatments using metabolomics and transcriptomics alongside microbial species diversity analyses to understand whether gut microbiomes can aid resilience under future climate change.

**Supplementary Information:**

The online version contains supplementary material available at 10.1186/s12866-026-05114-4.

## Introduction

Anthropogenic-mediated climate change in the West Antarctic Peninsula (WAP) poses a significant threat to marine biodiversity with Antarctic marine ectotherms amongst the least resilient organisms on Earth to change [62]. Although, it has long been acknowledged that healthy host microbiomes underpin resilient ecosystems, investigation of marine invertebrate microbiomes remains poor [83]. This is particularly true of the polar regions, where we have little data on host microbiomes and whether these could help support these highly vulnerable Antarctic marine invertebrates to adapt to future ocean warming [44]. Certainly, there is evidence from other regions of the globe, that such host-microbiome interactions can be beneficial. For example, microbes that colonise the digestive tracts of multicellular hosts can degrade complex dietary molecules, such as polysaccharides and provide essential micronutrients (e.g. cobalamin (vitamin B_12_) and nitrogen) [18, 57, 65]. These microbial populations are also dynamic and can restructure in response to the host’s environment and bolster the host’s ability to cope with seasonal change [43, 60, 71]. Indeed, beneficial bacterial strains are being promoted as promising biological remediation tools for enhancing tolerance to heat stress in corals [20].

To be able to answer vital questions on the potential role of microbial communities in mediating invertebrate host responses to change, increased research efforts to characterise Antarctic invertebrate microbiomes are required. In this respect, echinoderms present as tractable study species as they are phylogenetically diverse and encompass species from a range of trophic levels. They include primary consumers such as the holothurians (sea cucumbers), which rely on suspension or deposit feeding behaviour, secondary consumers such as the asteroids (star fish or sea stars), and omnivores such as the echinoids (sea urchins) and ophiuroids (brittle stars) [48]. Although echinoderms are amongst the most widely represented benthic groups in the Southern Ocean [14], to date, only a few studies have investigated microbiomes in this phylum. Previous studies include an investigation into the gut microbiome of the burrowing heart urchin, *Abatus agassizii*, on King George Island [70]. Núñez-Pons et al., [58] identified the potential significance of *Clostridium* (*Bacillota*) in individuals of *O. validus* that were healthy amongst a population experiencing an outbreak of Sea Star Wasting Disease (SSWD). Whilst Buschi et al., [8] identified *Rhodobacteriaceae* as core members of the *O. validus* microbiome, which they suggested may provide nutritional benefits for the host.

Given this paucity of Antarctic data and the fact that diet has been demonstrated as an important driver of microbiome structure in numerous invertebrates [30, 37] we investigated the gut microbiomes of six species of Antarctic echinoderms via 16S rRNA amplicon sequencing of the V3-V4 region. Analyses included measurements of alpha and beta-diversity alongside co-occurrence network analyses. Our study comprised three suspension feeders, the sea cucumbers *Cucumaria georgiana*, *Echinopsolus charcoti*, *Heterocucumis steineni* [16], a scavenger/predator, the sea star *Odontaster validus* [48], and two omnivores, the brittle star *Ophionotus victoriae* and the sea urchin *Sterechinus neumayeri* [6, 28]. Our aim was to identify how their gut microbiomes varied with feeding guild and also seasonally, between the highly productive austral summer and oligotrophic winter. Additionally, as warming in the WAP region will have major ramifications for Antarctic marine biodiversity we also assessed the response of primary consumers to the long-term outcomes of ocean warming by + 2 ˚C.

## Methods

### Sampling of animals 

Six species of average-sized adult Antarctic echinoderms (*O. validus NB: small v for validus*, *O. victoriae*,* E. charcoti*,* C. georgiana*, and *H. steineni* and *S. neumayeri*) were collected by SCUBA divers from South Cove, Ryder Bay, Adelaide Island, West Antarctic Peninsula (67°34 S, 68°08 W), at depths of 15 to 20 m. These animals were chosen as they are common in the near-shore marine environment around Rothera Research Station and are representative of a range of feeding guilds. The collection of animals took place across two years (2020–2021) and was not uniform across all the seasons, due to constraints set by extreme environmental conditions. *O. validus*,* O. victoriae*, and *H. steineni* were collected at three time-points: March 2020 (late austral summer), June 2020 (austral winter), and January 2021 (early austral summer), whilst *S. neumayeri*, *E. charcoti*, and *C. georgiana* were only sampled in June 2020 (winter) (Table 1). Upon collection, the animals were frozen at -20 ˚C and shipped to the UK. This preservation method ensured maintenance of integrity of the gut microbiomes [79].

**Table 1:** Summary of sample collections made in late summer (03/2020), winter (06/2020), and early summer (01/2021). The number of replicate samples for each species at the respective time-point is provided alongside their feeding strategies

Species	Natural sampling (total = 36)			Feeding strategy
	Late summer	Winter	Early summer	
Starfish				
* O. validus*	3	3	3	Generalist: scavenger, predator, omnivore
Brittle star				
* O. victoriae*	3	3	3	Detritivore, omnivore
Sea cucumbers				
* H. steineni*	3	3	3	Suspension feeders
* E. charcoti*		3		
* C. georgiana*		3		
Sea urchin				
* S. neumayeri*		3		Grazing omnivore

Additionally, during the first sampling point, (March 2020), extra samples (*n* = 88 in total) of averaged sized individuals of *H. steineni* (*n* = 28), *E. charcoti* (*n* = 35) and *C. georgiana* (*n* = 25) were collected. These individuals were not frozen upon collection but were returned to the aquarium in the Bonner laboratory at Rothera Antarctic Research Station, with care taken to keep them submerged throughout transportation, and subjected to a warming experiment (described below).

### Warming experiment

Prior to experimentation the animals were held for a maximum of five days to allow for recovery from any collection and handling stress in the flow-through aquarium at Rothera at ambient water temperatures. Afterwards, the animals were distributed randomly into five tanks. These comprised three tanks at ambient water temperature and two tanks which were kept at + 2 °C above ambient temperature (with in-tank heaters), with the + 2 °C tank temperatures tracking changes in the temperature of the ambient water inflow (Supplementary Data Table S1). Because all tanks were part of a flow-through aquarium system, they were all fed from the same water source and therefore technical replicates in the strict sense of the term (Table 2). This experiment lasted for six months. For 16 S rRNA amplicon sequencing, three specimens of each treatment and species were chosen for downstream microbiome analysis. These were selected from different tanks to control for tank effects.

**Table 2:** Summary of the six-month warming experiment. This experiment was started in March 2020 (late summer). The number of replicate samples for each species at the respective time-point is provided, with the number of samples selected from each tank for 16 S rRNA amplicon sequencing in this study provided in brackets (*n* = 18 samples in total)

Species	+ 2 ˚C		Control		
	Tank 1	Tank 2	Tank 3	Tank 4	Tank 5
Sea cucumbers					
* H. steineni*	4 (2)	12 (1)	6 (1)	1 (1)	5 (1)
* E. charcoti*	13 (2)	11 (1)	6 (1)	1 (1)	4 (1)
* C. georgiana*	6 (2)	8 (1)	4 (1)	2 (1)	5 (1)

The animals were held under control and experimental conditions described below in the Bonner laboratory flow-through aquarium for six months (with ambient water temperatures ranging from 1.047 °C in March 2020 to -1.557 in August 2020, at the end of the experiment). Lighting conditions in the aquarium were set at 12 h of light and dark throughout the experimental period and kept identical between the warming treatments and ambient control treatments. Aeration was applied. Ambient control animals were held at temperatures experienced in Ryder Bay, whilst the warming animals had tank temperatures raised by 0.2 ˚C Day^− 1^ until reaching 2 ˚C ± 0.2 ˚C above ambient, using standard aquarium heated probes. All animals were kept without feeding, although the flow-through system likely permits the passage of small phytoplankton species present in Ryder Bay into the aquaria, it is likely that the larger species are filtered out. It is also known that phytoplankton availability in natural conditions drastically declines from late summer and food supplies are generally insufficient to sustain feeding in *H. steineni* and *C. georgiana*, between the months of May and October [16]. At the end of the experimental period, the animals were frozen at -20 ˚C, prior to storage and shipping to the UK.

### DNA extraction

DNA was extracted from gut samples of all 54 frozen animal specimens summarised in Tables 1 and 2. The gut tissue was aseptically dissected. Subsequent DNA extraction and PCR amplifications were also conducted in laminar flow hoods to prevent contamination. Additionally, dissection tools were UV sterilised and chemically treated prior to use. DNA extractions were performed using the DNeasy^®^ PowerBiofilm^®^ kit (Qiagen) according to the manufacturer’s instructions with 230–270 mg of tissue added to each extraction kit, dependent on the size of gut tissue available in the invertebrate specimen. DNA quality and integrity were assessed using the Nanodrop One™ (ThermoScientific) and DNA quantity was measured using a Qubit™ 4 fluorometer (Invitrogen by Thermo Fisher Scientific) using the Invitrogen™ Qubit™ HS DNA assay kit. DNA extraction blanks were performed, whereby the extraction procedure was performed without the input of sample and quantified alongside the samples.

### 16S rRNA amplicon sequencing 

The 16S rRNA gene (V3-V4 region) of microbial communities present in the DNA extracts was amplified using the primers 341F (CCTAYGGGRBGCASCAG) and 806R (GGACTACNNGGGTATCTAAT) [88]. These primers were used in a previous microbial project in the lab and allowed comparisons to be made between the two datasets [34]. PCR amplification was performed using Q5™ (New England Biolabs) according to manufacturer’s instructions in 25 µl reactions with 1–2 µl of sample DNA. PCR conditions were as follows: 30 s at 98 ˚C, followed by 35 cycles of 98 ˚C for 10 s, 57 ˚C for 30 s, and 72 ˚C for 30 s, and a final extension step of 72 ˚C for 3 min. DNA extraction blanks were amplified alongside the sample DNA extractions and all PCR products were visualised on a 1.5% agarose gel. The DNA extraction blanks did not display amplification and successful PCR amplicons of the 54 specimens were kept at -20 ˚C prior to being sent to Novogene UK (Cambridge) for amplicon sequencing using ligation. To prevent PCR inhibition, Novogene performed PCR-free amplification and 16S rRNA gene amplicon sequencing using the Illumina NovaSeq 6000 platform (2 × 250 bp). The total outputs of DNA concentrations of 28 samples were below 100 ng after library preparation by Novogene and these libraries were pooled together prior to sequencing. During the bioinformatic analysis, Cumulative-sum scaling (CSS) normalisation steps were later performed to control for the effects of this pooling [61]. It should be noted that DNA negative controls were processed but not sequenced in this study due to a lack of template amplification. This is a limitation as negative controls offer an opportunity to observe laboratory contamination that has arisen during sample handling, DNA extraction, or amplification, even when amplicon yield is low [69]. These contaminants would be expected to create a ubiquitous or batched background signal that would interfere with the interpretation of bacterial composition and vary in relative abundance based on sample biomass. Despite lacking these negative controls, Decontam detected and removed many taxa with this pattern (such as Afipia, Cutibacterium, Sphingobium) which are previously reported as common sources of exogenous DNA contamination [19]. The parameters of the package are described in the bioinformatic analysis pipeline below.

### Bioinformatic analysis of amplicon sequencing

Following sequencing, Novogene provided sequencing paired end reads that had been demultiplexed. Primers and adaptors were trimmed from the reads using the ‘*QIIME2*’ 2024.10 Amplicon Distribution [4] and Cutadapt 4.9 with Python 3.10.14 plugin [46]. Afterwards, the ‘*DADA2*’ version 1.30.0. plugin was run using R version 4.3.3, ‘*Rcpp*’ version 1.0.13.1 and ‘*RcppParallel*’ version 5.1.9 [9] was used to denoise and dereplicate the sequencing reads and produce amplicon sequencing variants (ASVs) using the trimming parameters of 220 bp for both forward and reverse reads. Taxonomic classification of ASVs was performed using the primer-specific trained Naïve Bayes taxonomic classifiers with the SILVA Release 138.2 [67, 87]. The paired sequences were merged using the pipeline before producing the ASV table (all accessed on the 2nd of February 2025). Non-bacterial ASVs, singletons, and ASVs identified as chloroplast or *Cyanobacterota* were removed prior to diversity and statistical analysis, resulting in a total of 7,381 ASVs. The removal of archaeal ASVs and *Cyanobacterota* was due to known biases of the prokaryotic universal 16S primers (in this case the V3-V4 region) in identifying these groups [42, 77] and significantly low abundances of reads that limited biological interpretability. Bioinformatic analyses were conducted according to previously published methods [12]. Potential contaminant ASVs were identified using the isContaminant function from the package ‘decontam’ with a threshold of 0.5 and batch mode enabled [19]. DNA concentration data from Qubit measurements was used for the ‘frequency’ method, as negative controls were not sequenced in this study due to a lack of template amplification. ASVs identified as potential contaminants were filtered prior to all downstream analysis. ASVs that shared taxonomy with potential contaminants but were not flagged by ‘decontam’ were not removed due to their low relative abundances and limited taxonomic resolution.

CSS was used to normalise ASV counts and was performed using the ‘metagenomeSeq’ package [61]. CSS was selected as it is specifically designed for sparse, zero-inflated microbiome count data and accounts for differences in sequencing depth without discarding reads. In contrast to rarefaction, which reduces statistical power through subsampling [50], CSS retains all observations while mitigating biases associated with uneven library sizes. Alternative approaches such as DESeq2 [45] were not applied as they rely on negative binomial distribution assumptions and library size normalisation developed for RNA-seq data, which may not be appropriate for highly sparse and compositional amplicon datasets. Comparative studies have shown differential abundance results can vary substantially depending on the normalisation method applied [55]. Furthermore, comparative assessments of microbiome normalisation methods indicate that scaling-based approaches, such as CSS, perform robustly across datasets with varying sequencing depth and sparsity [82].

Each experiment’s ASV table was normalised separately because the datasets differed in species composition, sequencing batches and experimental design. Joint normalisation across all samples could introduce batch effects and overly conservative scaling due to differences in community structure and sequencing depth, potentially masking biologically meaningful variation. Separate normalisation therefore preserves within-experiment comparability while reducing cross experiment bias. The Culturome pipeline [90] was used to identify cultured representatives from a previous study on the same species [34], that shared a 100% 16S rRNA gene similarity with the ASV sequences from the warming experiment. Alpha diversity metrics of the normalised ASV data were calculated in the package ‘*Phyloseq*’ [49]. Two metrics of alpha diversity were included; ‘Shannon diversity’, an index which is calculated based on richness and evenness and ‘observed ASVs’, a simple measure of richness representing the total count of unique ASVs in each sample following CSS normalisation [39]. Beta-diversity metrics were calculated using Bray-Curtis dissimilarity in the R package ‘vegan’. The dissimilarity distances were visualised using non-dimensional scaling (NMDS). A summary of packages which were run in R and additional packages used in plotting the figures is provided in Supplementary Data Table S2.

### Statistical analysis

Statistical analyses were carried out using SigmaPlot version 15.0, Build 15.1.1.26. Normally distributed data, based on Shapiro-Wilks test and equal variance test (Brown-Forsythe) were tested for significance using one-way ANOVA followed by Tukey’s pairwise comparisons. Power calculations of the one-way ANOVA analysis tests were performed in SigmaPlot, to verify whether the desired power was above the recommended value of 0.8 [41]. In the warming experiment, normally distributed data (observed ASVs, Shannon) were compared using Student’s t-tests, while non-normal data were compared with Mann-Whitney U tests. PERMANOVA analysis to test for differences in community structure in beta-diversity analysis was conducted using the ‘adonis2’ function in the R package ‘vegan’. The homogeneity of group dispersions in Bray-Curtis was tested using the ‘betadisper’ function in vegan. Statistical significance was denoted as a *p* value < 0.05.

### Co-occurrence network analysis

Whilst alpha and beta metrics reveal differences in bacterial community richness, evenness, and community composition they do not consider relationships between taxa. Therefore, network analyses were conducted to explore interactions between taxa across trophic levels, seasons, and simulated warming [36]. The Network Construction and comparison for Microbiome data (NetCoMi) R package (version 1.2.0) [64] was used to perform network analysis on the omnivores (*O. victoriae* and *S. neumayeri*) and suspension feeders (*C. georgiana*, *E. charcoti*, and *H. steineni*) during winter, three echinoderms (*H. steineni*, *O. validus*, *O. victoriae*) during the seasonal microbiome study, and the warming vs. ambient microbiomes of each of the suspension feeders during the warming microbiome study. Prior to network construction, singletons and ASVs below 1% relative abundance were filtered out. The ‘netConstruct’ function, with SparCC [29] for clustering, and dissimilarities were transformed using the “signed” method. The ‘netAnalyze’ function was used for network construction. The default parameters were used, except weightDeg was set to TRUE, prior to visualisation.

## Results

Amplicon sequencing of the 16S rRNA gene (V3-V4 region) produced 159,725,002 high-quality reads, which were assembled into 7,072 ASVs, after the removal of Archaea, singletons, chloroplast, *Cyanobacteriota* and potential contaminants. Details of raw, filtered, and normalised read counts associated with each specimen are provided in Supplementary Data Tables S3-S5. Rarefaction curves of all 54 sequenced samples have been included to supplementary material (see Supplementary Data Figure S1A-C). All samples reach a plateau of observed ASVs with increasing sequencing depth, indicating that sequencing efforts were sufficient to capture microbial diversity. In total, 309 ASVs were identified as potential contaminants (Supplementary Data Table S6). The contaminant list included some strains of bacteria commonly identified as DNA extraction kit and laboratory contaminants, such as *Cutibacterium*, *Afipia*, and *Sphingobium* [21, 69]. The list may not be exhaustive or potentially include false positives. Furthermore, to carefully assess the potential impact of sample pooling prior to sequencing we compared Shannon diversity and observed ASVs with pooled versus non-pooled groups (see Supplementary Data Figure S2). Overall, pooling did not bias Shannon diversity or observed ASVs nor change the main conclusions of the paper. Nevertheless, the assessment above and evidence from the rarefaction curves (Supplementary Data Figure S1) showing that all samples reached a plateau for both pooled and non-pooled samples suggest that pooling did not affect the capture of the majority of microbial diversity, nor bias the overall conclusions of the paper.

The 54 collected specimens were separated into three distinct studies (winter microbiome, seasonal microbiome, and warming microbiome). The gut microbial communities of the animals in each of these three experiments were compared by analysing metrics of alpha-and beta-diversity, as well as taxonomic composition.

### Winter microbiomes

**Fig. 1:**
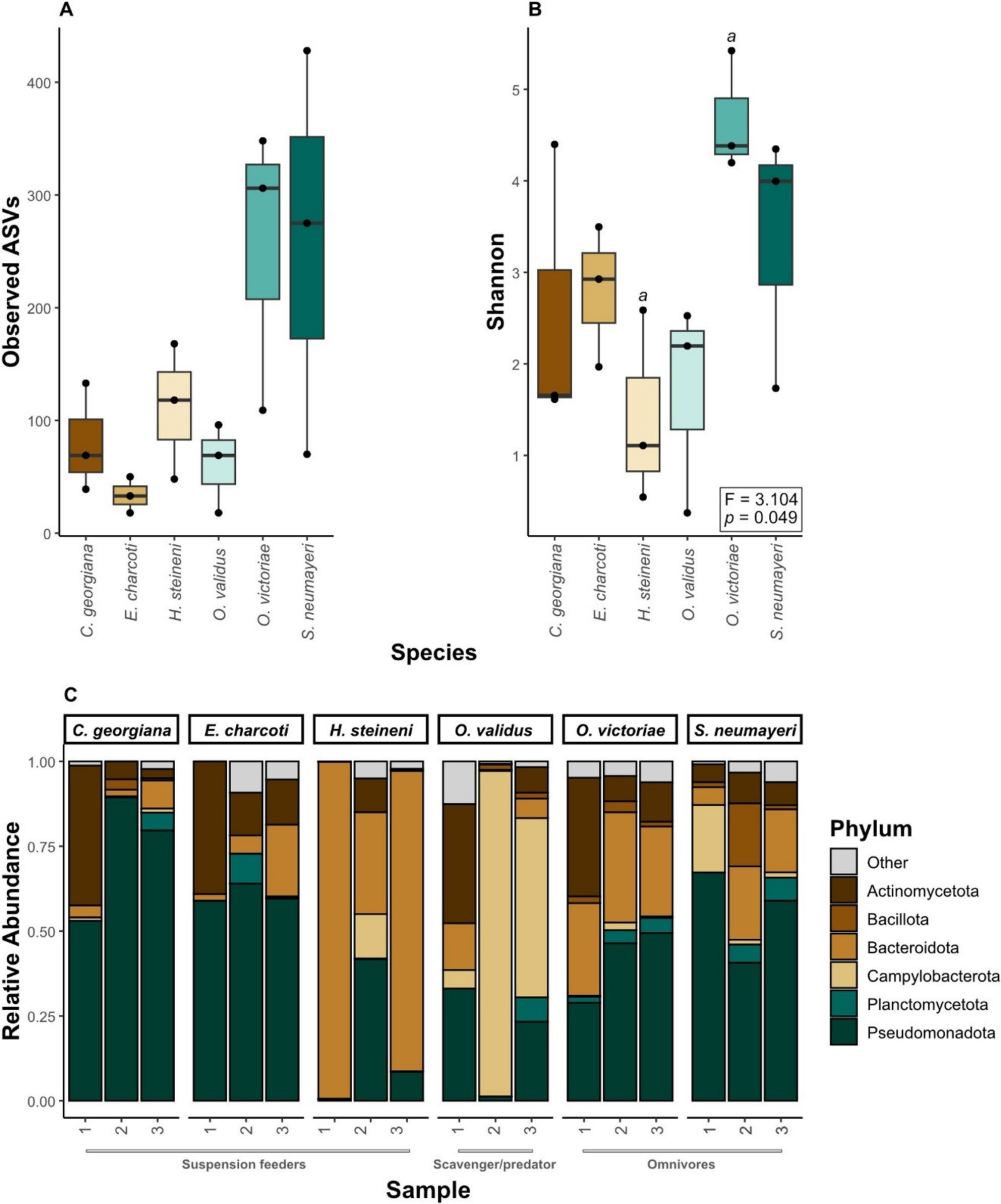
Alpha diversity metrics and taxonomic composition of winter gut microbiomes (**A**) Observed ASVs and (**B**) Shannon diversity after CSS normalisation. Lower case letters denote comparisons that were significantly different (*p* < 0.05) in post-hoc Tukey comparisons that followed a significant one-way ANOVA (indicated in box). The box plots display the medians (shown as black line), lower and upper quartiles, and maximum and minimum values (shown by whiskers). **C** The relative abundances of bacteria are provided at phylum level based on 16 S rRNA gene V3-V4 region sequence identities. Only bacterial phyla that presented median relative abundance values greater than 1% within each species are shown, whilst those that fell below this criterion were grouped into ‘Other’

All six echinoderms (*C. georgiana*, *E. charcoti*, *H. steineni*, *O. validus*, *O. victoriae*, and *S. neumayeri*) were sampled during winter at a single time point. The results of a global comparison for alpha diversity amongst species were borderline for both observed ASVs and Shannon diversity (One-way ANOVA, *p* = 0.050 and 0.049, respectively). Bacterial diversity displayed peak values in the omnivores, *O. victoriae* and *S. neumayeri*, with minimum values amongst the sea cucumbers and *O. validus* (Fig. 1A-B), nevertheless, the power calculations of both alpha-diversity tests were below the desired threshold of 0.8, at 0.492 and 0.496, respectively. However, in the beta-diversity analyses, PERMANOVA analysis of Bray-Curtis dissimilarity distances showed significant variation across all six echinoderm species in winter (*p* = 0.001) indicating a correlation between bacterial diversity patterns with species and feeding guild. NMDS visualisations across the six echinoderms placed the omnivores *O. victoriae* and *S. neumayeri* in overlapping clusters, correlating with their similarity in the alpha-diversity metric results (Supplementary Data Figure S3). The three sea cucumbers and *O. validus* were more divergent in their alpha-diversity metrics compared with the omnivores (Supplementary Data Figure S3). Interestingly, only a single ASV belonging to the genus *Knoellia* was identified as shared between all the suspension feeders (Supplementary Data Figure S4A), whilst the omnivores, *O. victoriae* and *S. neumayeri* shared the highest number of overlapping ASVs, accounting for 11% of ASVs amongst the scavenger/predator and omnivore comparison (Supplementary Data Figure S4B). There were virtually no shared ASVs (< 1%) between either of the omnivores *O. victoriae* and *S. neumayeri* and the predator/scavenger *O. validus* (Supplementary Data, Figure S4B). Non-significant PERMDISP tests established that the PERMOVA results were due to difference in community composition and not unequal dispersions of variance. Full details of statistical analysis performed on alpha- and beta-diversity metrics are provided in Supplementary Data Tables S7-S8.

In terms of the taxonomic composition of the winter gut microbiomes, six bacterial phyla predominated, although there was high variability between individuals of each species. The median level for this analysis was set at greater than 1% relative abundance in each host species, with the median selected to ensure phyla were greater than 1% in two samples or more (Fig. 1C). Of these six phyla, *Pseudomonadota* dominated the two sea cucumbers, *C. georgiana* (53–89%) and *E. charcoti* (59–64%), and were present in high relative abundances (mean: 29–67%) in the other species, except for *H. steineni* which varied from 0.4 to 42%. *Bacteroidota* were the second most common phyla and, in particular, dominated the community composition of *H. steineni* (30–99%). *Campylobacterota* dominated the community composition of two individuals of *O. validus* (up to 96%), whilst presence of this phylum in the third individual of *O. validus* was much lower at only 5%. *Campylobacterota* were at generally lower relative abundances in all other species (0–20%) and were not present in *E. charcoti*. Other common phyla across all six echinoderms were *Actinomycetota* (total mean: 13%), *Bacillota* (total mean: 2%), and *Planctomycetota* (total mean: 2%). At class level, *Alphaproteobacteria* were dominant members of *Pseudomonadota*, accounting for over 90% of ASVs from this phylum, in all but one sample of the six echinoderms (Supplementary Data Figure S5). *Bacteroidia* and *Campylobacteria* were representative classes of the phyla, *Bacteroidota* and *Campylobacterota*. The classes *Acidimicrobiia* and *Actinobacteria* were dominant representatives of *Actinomycetota* (Supplementary Data Figure S5).

**Fig. 2:**
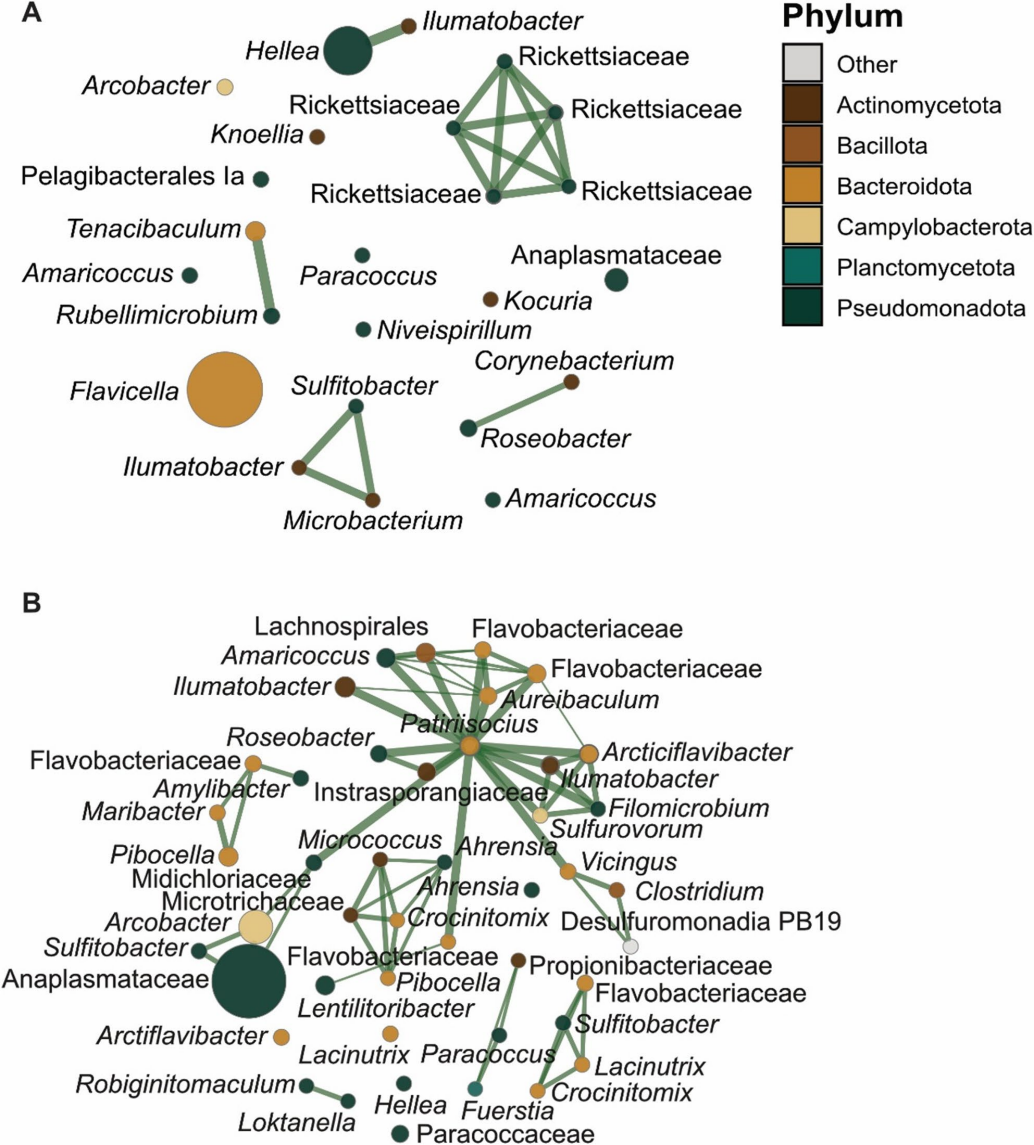
Co-occurrence network analysis of (**A**) sea cucumbers; *C. georgiana*, *H. steineni*, and *E. charcoti* and (**B**) omnivores, *O. victoriae* and *S. neumayeri*. The edge weights represent non-negative similarities. The node size represents the normalised counts

Network analysis of the microbiomes was conducted at ASV level, to identify any potential interactions associated with feeding guild. In the suspension feeding sea cucumbers this revealed limited patterns of co-occurrence in winter (Fig. 2A), whereas in the omnivore (*O. victoriae* and *S. neumayeri*) microbiomes, positive correlations were identified both within and between phyla. In particular, the *Patiriisocius* ASV (order *Flavobacteriales)* was identified as the hub for a multi-phylum cluster comprising members of the *Actinomycetota*,* Bacillota*,* Bacteroidota*,* Campylobacterota* and *Pseudomonadota* (Fig. 2B).

### Seasonal microbiomes

Three echinoderms (*H. steineni*, *O. validus* and *O. victoriae*) were sampled during the austral late summer (2020), winter (2020/21) and early summer (2021). It should be noted that the two summer samplings occurred in different years and months. Late summer (March) which is generally towards the end of the phytoplankton bloom and early summer (January) in the peak phytoplankton bloom, subject to interannual variability [15]. Chlorophyll data (which is used as a proxy for phytoplankton abundance) was obtained from our long-term monitoring site at Rothera research station for March 2020 and January 2021 (https://www.bas.ac.uk/project/rats/). These data showed that in March 2020 the phytoplankton bloom was diminishing with chlorophyll concentrations decreasing from 3.523 mg m^− 3^ to 0.446 mg m^− 3^ over the month, whereas in January 2021 the chlorophyll concentrations were on the increase from 3.491 mg m^− 3^ to 8.247 mg m^− 3^ over the month, which could clearly impact the composition of the gut microbiomes. The phytoplankton bloom also varies annually [81] and therefore prior feeding history could also play a role in gut bacterial communities, as determined by single point time samplings detailed here.

**Fig. 3:**
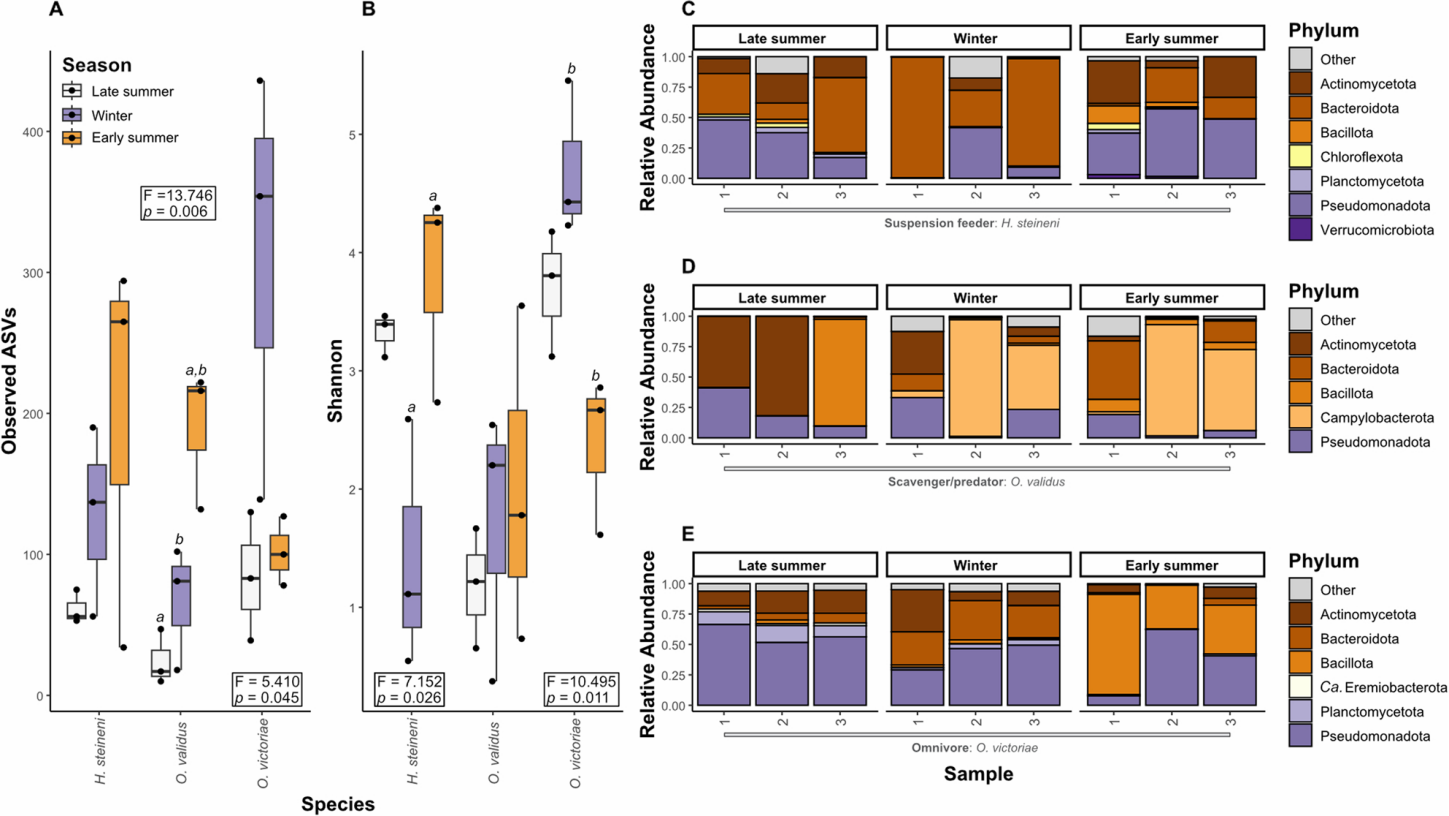
Alpha diversity metrics and taxonomic composition of seasonal gut microbiomes (**A**) Observed ASVs and (**B**) Shannon diversity after CSS normalisation. Lower case letters denote comparisons that were significantly different (*p* < 0.05) in post-hoc Tukey comparisons that followed a significant one-way ANOVA (indicated in box). The box plots display the medians (shown as black line), lower and upper quartiles, and maximum and minimum values (shown by whiskers). The relative abundances of bacteria from (**C**) *H. steineni*, (**D**) *O. validus*, and (**E**) *O. victoriae* are provided at phylum level based on 16 S rRNA gene V3-V4 region sequence identities. Only bacterial phyla that presented median relative abundance values greater than 1% within each species are shown, whilst those that fell below this criterion were grouped into ‘Other’. Please note that the *Candidatus* phylum *Eremiobacterota* is now reclassified as phylum *Vulcanimicrobiota*

There were seasonal changes in alpha-diversity with regard to observed ASVs and Shannon diversity in all three species (Fig. 3A-B). These changes were pronounced in the omnivore *O. victoriae* and the suspension feeder *H. steineni* whereby Shannon diversity in *O. victoriae* decreased from winter to early summer (One-way ANOVA: *p* = 0.011, power = 0.850) whilst *H. steineni* Shannon diversity followed the opposite trend and increased towards early summer (One-way ANOVA: *p* = 0.026, with near desirable power of 0.672). Shannon diversity values for *O. victoriae* were 1.98-fold higher in winter as compared to early summer (Tukey, *p* = 0.009). In contrast, Shannon diversity for *H. steineni* were 2.67-fold higher in early summer as compared to winter (Tukey, *p* = 0.028). Whilst observed ASVs for *O. victoriae* varied seasonally (one-way ANOVA: *p* = 0.045), across sample variation was much greater in winter and only marginally higher as compared to late summer (Tukey, *p* = 0.057), with a lower power value of 0.526. The *O. validus* (scavenger/predator) microbiome increased significantly in observed ASVs from late summer to early summer (One-way ANOVA: *p* = 0.006, power = 0.936), with 7.70-fold more observed ASVs in early summer compared to late summer (Tukey, *p* = 0.006) and 2.83-fold in winter (Tukey, *p* = 0.022). No significant differences were observed in Shannon diversity across seasons for *O. validus*. Full details of statistical analysis of alpha-diversity metrics in the seasonal microbiome experiment are provided in Supplementary Data Table S9.

NMDS of Bray-Curtis dissimilarity distances measuring beta-diversity of all three echinoderms across the season clustered the species in distinct groups (Supplementary Data Figure S6). When all three species and seasons were analysed together, PERMANOVA analysis resulted in significant variations on the basis of species (*p* = 0.001) and season (*p* = 0.024). The three sets of data from each of the echinoderms were then separately analysed by species to identify if a seasonal effect was present in the beta-diversity metrics. Following this, PERMANOVA analysis revealed significant effects of season on Bray-Curtis dissimilarity distances of *O. victoriae* (*p* = 0.004), *H. steineni* (*p* = 0.016), with a higher *r*^2^ from season alone in *O. victoriae* (Supplementary Data Figure S6B-D and Table S10), but no significant seasonal differences in *O. validus*. The variability of Bray-Curtis dissimilarity distances amongst replicates of *O. validus* and *H. steineni* was lowest during the late summer period. No significant differences were observed in PERMDISP tests across species and seasons. Full details of statistical analysis conducted on beta-diversity analysis are detailed in Supplementary Data, Table S10.

A low percentage of total ASVs were shared between the three echinoderms across the seasonal microbiome (1%) (Supplementary Data Figure S7A), which was mirrored when shared ASVs were analysed seasonally within each species (Supplementary Data Figure S7B, C and D) at < 1%.

Taxonomic analysis of the bacterial phyla in this seasonal experiment showed low abundances with only nine accounting for most of the diversity (greater than 1% across each species). The relative abundances of some of these phyla varied seasonally (Fig. 3C-E). *Pseudomonadota* were represented in all the microbiomes of *O. validus*, *O. victoriae*, and *H. steineni*. Levels of *Bacteroidota* decreased from ~ 29% to ~ 2%, between the winter and early summer microbiome of *O. victoriae*. Similarly, *Bacteroidota* decreased from ~ 73% to 16%, between the winter and early microbiome of *H. steineni*. *Bacillota* groups were most prominent in the early summer microbiome (mean: ~53%) of *O. victoriae* compared to its late summer (mean: ~1%) and winter (~ 2%) microbiome (Fig. 3E). Additional abundant phyla were *Campylobacterota*, particularly in *O. validus* varying from 2% to 95% in early summer and winter but not present in late summer (Fig. 3D). Of note, the reduced spread of diversity displayed in *O. validus* during late summer compared to early summer and winter coincided with a lack of detection of *Campylobacterota* during this period. *Actinomycetota* and *Planctomycetota* were also present at notable relative abundances across all three echinoderms, with taxa such as *Chloroflexota* and *Verrucimicrobiota* only being present at a median greater than 1% in *H. steineni* (Fig. 3C) and *Candidatus* phylum *Eremiobacterota* (now reclassified as phylum *Vulcanimicrobiota*) in *O. victoriae*. Interestingly, this phylum was only present at > 1% in the late summer microbiome of *O. victoriae*, and present at low levels (< 0.5%) in a single winter sample of *O. victoriae* and a single early summer sample of *H. steineni.* As observed in the winter microbiome, *Alphaproteobacteria* remained dominant class member along with representatives of *Pseudomonadota*, although seasonal oscillations were observed (Supplementary Data Figure S8). Relative abundances of *Gammaproteobacteria* in the gut microbiome of *O. victoriae* varied from a mean of 2.5% during the winter to < 0.5% in early summer and late summer. The classes *Bacilli* and *Clostridia* were dominant representatives of *Bacillota*. *Bacilli* were only present at median relative abundances > 1% in *O. validus*, whilst *Clostridia* were identified in all three species, with highest abundances in *O. victoriae*, early summer (mean: 42%). The classes *Acidimicrobiia* and *Actinobacteria* were dominant representatives of *Actinomycetota*. *Acidimicrobiia* were predominantly found in *H. steineni* and *O. victoriae*.

**Fig. 4:**
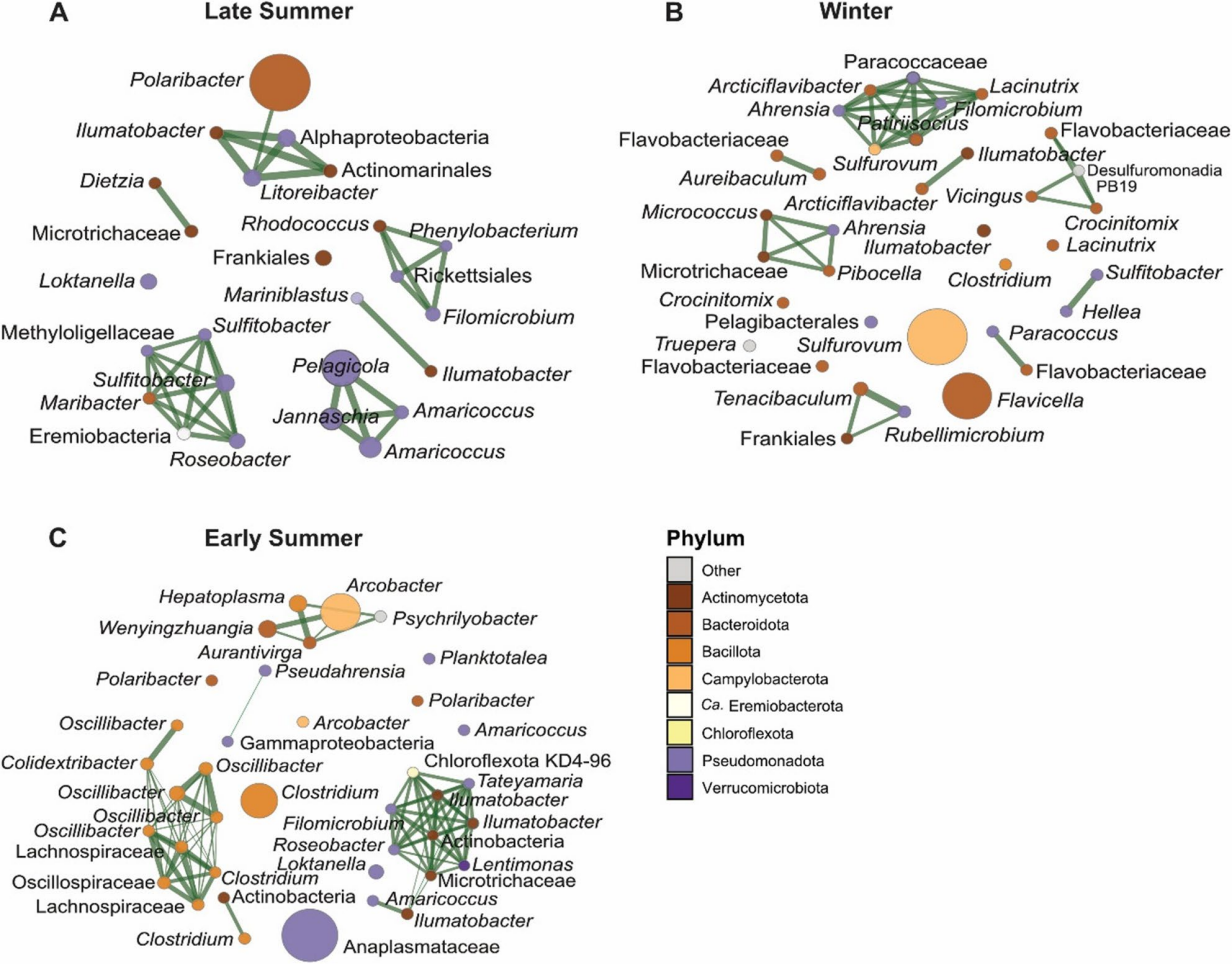
**C**o-occurrence network analysis of *H. steineni*, *O. validus*, and *O. victoriae* in (**A**) late summer, (**B**) winter, and (**C**) early summer. The edge weights represent non-negative similarities. The node size represents the normalised counts. The *Candidatus* phylum Eremiobacterota is now reclassified as phylum *Vulcanimicrobiota*

Co-occurrence analysis of the seasonal microbiome for the three host species yielded several correlation clusters unique to each season and illustrates the dynamic nature of the gut microbiome (Fig. 4). These include single-phylum clusters such as the *Bacillota* seen in early summer (Fig. 4C), or multi-phylum clusters such as that featuring *Patiriisocius* in winter (Fig. 4B). However, the networks also imply scarce relationships exist between the most abundant taxa at each timepoint (*Polaribacter* in late summer, *Sulfurovum* and *Flavicella* in winter, *Anaplasmataceae* and *Arcobacter* in early summer). Although network analysis cannot provide evidence for the role of these bacteria in seasonal responses, co-associated groups could be targets for future functional investigations.

## Warming experiment microbiome

**Fig. 5:**
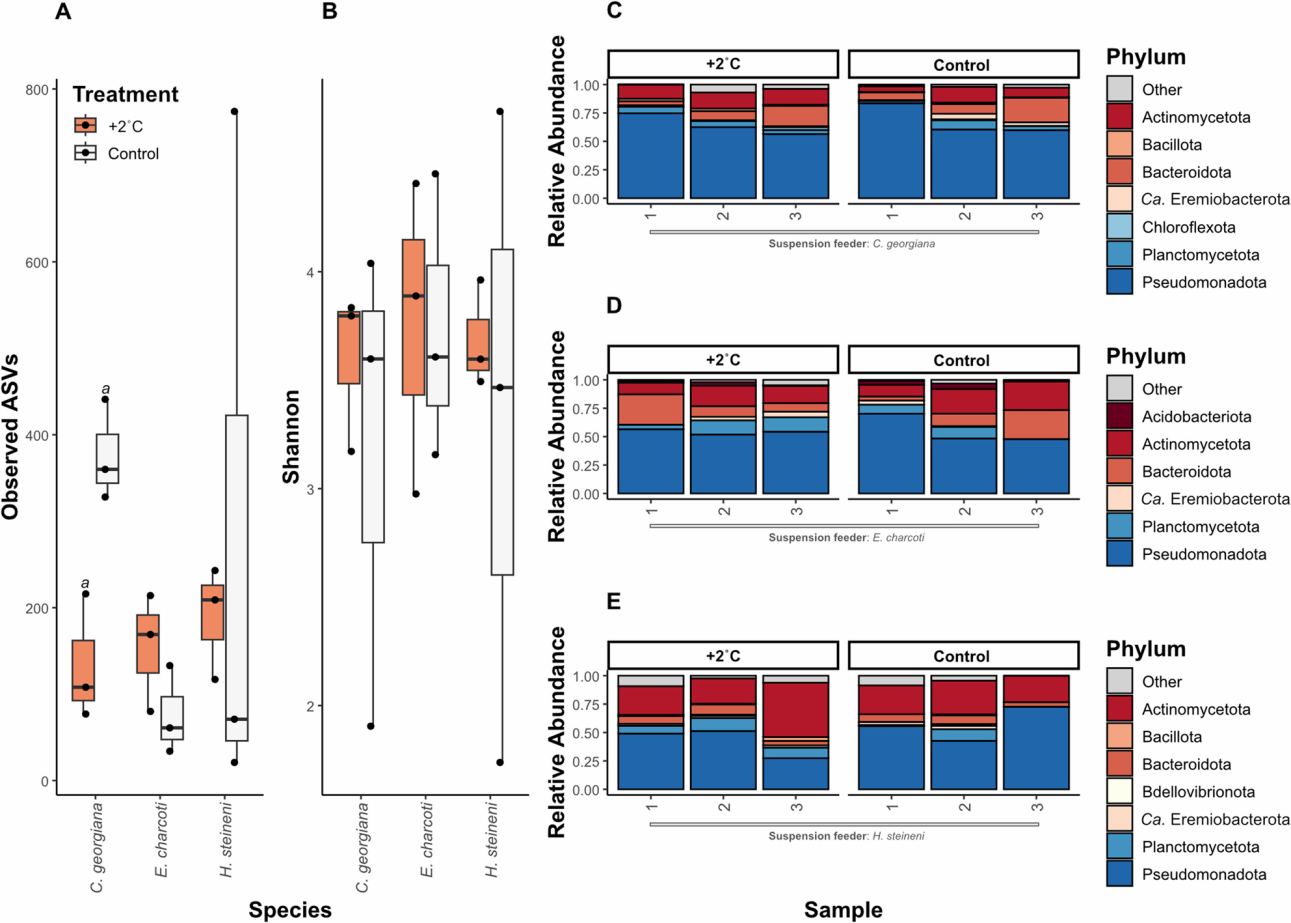
Alpha diversity metrics and taxonomic composition of *C. georgiana*,* E. charcoti*,* and H. steineni* following a six-month warming experiment. **A** Observed ASVs and (**B**) Shannon diversity after CSS normalisation. Lower case letters denote comparisons that were significantly different (*P* < 0.05) in post-hoc Tukey comparisons that followed a significant one-way ANOVA (indicated in box). The box plots display the medians (shown as black line), lower and upper quartiles, and maximum and minimum values (shown by whiskers). Full details of statistical analysis performed on alpha-diversity metrics are provided in Supplementary Data Table S11. The relative abundances of bacteria from (**C**) *C. georgiana* (**D**) *E. charcoti*, and (**E**) *H. steineni* are provided at phylum level based on 16 S rRNA gene V3-V4 region sequence identities. Only bacterial phyla that presented median relative abundance values greater than 1% within each species are shown, whilst those that fell below this criterion were grouped into ‘Other’. The *Candidatus* phylum Eremiobacterota is now reclassified as phylum *Vulcanimicrobiota*

Three sea cucumbers (*C. georgiana*, *E. charcoti*, and *H. steineni*) were collected during the late summer season and subjected to a six-month + 2 °C acclimation experiment to investigate the effect of warming on their microbiomes. Alpha-diversity analyses of observed ASVs and Shannon diversity were similar between sea cucumbers that had been kept at ambient temperatures and those that had been subjected to + 2 ˚C (Figs. 5A and B). The exception was for observed ASVs in *C. georgiana* (Fig. 5A), where observed ASVs were significantly higher in the control treatment. The Shannon alpha-diversity metrics were consistent across the species. There were no statistically significant differences in observed ASVs, Shannon diversity and dominance between the gut microbiome at + 2 ˚C compared to ambient temperatures in *E. charcoti* and *H. steineni*. In *C. georgiana*, there was no significant difference in Shannon diversity but significantly lower observed ASVs were recorded in warming conditions (Student’s t-test, *p* = 0.0108) (Supplementary Data Table S11).

The beta-diversity NMDS visualisations of Bray-Curtis dissimilarity distances showed clusters of the three sea cucumbers according to their species (Supplementary Data Figure S9A), with a significant effect of species (PERMANOVA, *p* = 0.001). No significant effect of warming was observed when all three species and warming conditions were analysed together and the effects of species accounted for (PERMANOVA, *p* = 0.132). The *r*^2^ value of the PERMANOVA of the combined effects of species and treatment was higher (0.351) than the effect of species alone (0.189). PERMANOVA analysis performed on individual species found no significant effect of warming (Supplementary Data Figure S9B-D). No significant differences were observed in PERMDISP tests across species and treatments. Full details of statistical analysis conducted on beta-diversity analysis are detailed in Supplementary Data Table S12. The number of shared ASVs between all three sea cucumbers was higher in the warming experiment (3%) compared to the natural winter microbiome (only one ASV) (Supplementary Data Figure S10). Interestingly, only a low percentage of ASVs were overlapping between control and warming conditions of all three sea cucumbers (10–14%), but as shown in Figs. 5A-B, there was high variability amongst replicates.

Taxonomic analyses showed low abundance for most phyla with eight accounting for the majority of the diversity (Fig. 5C-E). Despite high variability in numbers of distinct taxa, the taxonomic composition of the three species did not vary significantly between control and warming conditions (Fig. 5C-D). *Pseudomonadota* dominated all treatments and species with means ranging from 48% to 63%. *Actinomycetota* were the next most abundant (total mean: 19%) and *Bacteroidota* (mean: 11%). *Acidobacteriota* were not identified in *H. steineni*, found at very low abundance (< 0.1%) in one replicate of *C. georgiana*, but slightly greater than 1% abundance in *E. charcoti* at control and warming conditions. *Chloroflexota* were only present at a median greater than 1% in *C. georgiana* and not *H. steineni.* Notably, *Candidatus* phylum Eremiobacterota (now *Vulcanimicrobiota*) were identified at greater than 1% across all three sea cucumber species. Analysis of taxonomic composition at class level revealed similar conditions to those seen in natural winter conditions, whereby, *Alphaproteobacteria* accounted for more than 91% of *Pseudomonadota* members (Supplementary Data Figure S11). Nevertheless, higher levels of *Gammaproteobacteria* were observed in *H. steineni* and *E. charcoti* than *C. georgiana*, although there was some level of sample variability. The *Acidimicrobiia* and *Actinobacteria* were the most common members of *Actinomycetota*.

**Fig. 6:**
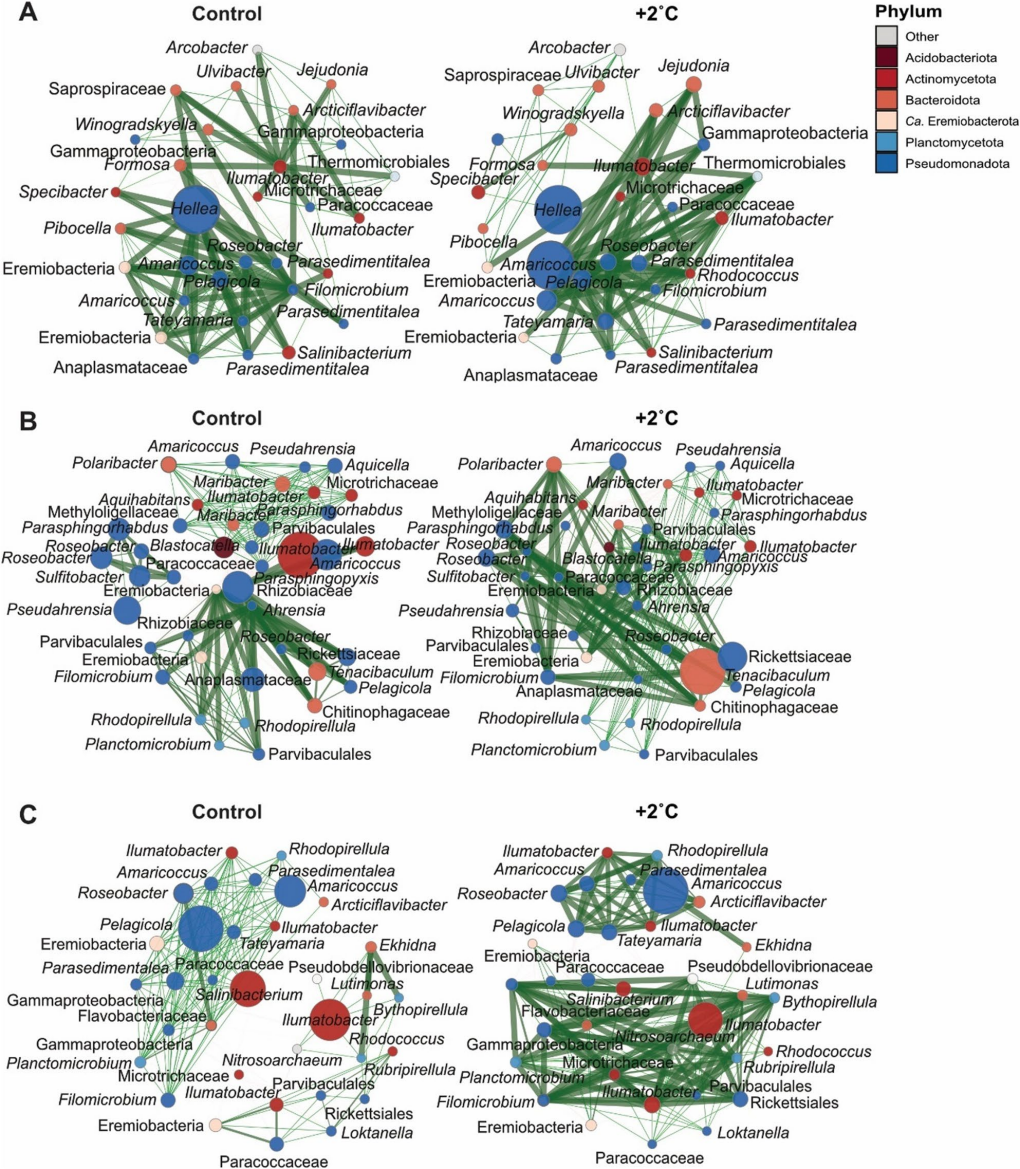
Co-occurrence network analysis of (**A**) *C. georgiana*, (**B**) *E. charcoti*, and (**C**) *H. steineni* in control and warming conditions. The edge weights represent non-negative similarities. The node size represents the normalised counts. Please note that the *Candidatus* phylum *Eremiobacterota* is now reclassified as phylum *Vulcanimicrobiota* and the class *Eremiobacteria* is now reclassified to *Vulcanimicrobiia*

As expected for samples from a controlled environment, co-occurrence networks illustrate high connectivity due to a greater degree of similarity between the replicates of each host species (Fig. 6). The most abundant ASVs differ between hosts, and in the case of *Hellea* in *C. georgiana* the dominant genus was also unique to the host species. A restructuring of associations is apparent in the warmed groups, driven in part by the increased representation across all host species of specific *Pseudomonadota* members, including genera *Amaricoccus*, *Tateyamaria*, and *Parasedimentitalea*.

## Discussion

Characterising microbial communities in keystone invertebrate species of the WAP is essential groundwork for understanding what role they may play in aiding the adaptation of these vulnerable species to changing climatic conditions. Here, we assessed the gut microbiome of six echinoderm species using metagenomic 16 S rRNA amplicon sequencing to provide a baseline understanding of the impacts that feeding guild, seasonality and warming could have on these gut microbial communities. Not surprisingly such composition was largely host-specific and correlated with the diets of the invertebrates, with highest alpha diversity found in the omnivores *O. victoriae* and *S. neumayeri.* Seasonality also had a significant effect on gut bacterial diversity particularly in the suspension feeders, although long-term warming to + 2 °C appeared to have little impact. Unfortunately, due to cost limitations, our analyses were restricted to *n* = 3 replicates per species/timepoint. Variation in any measures of wild species is always high (as evidenced in this study by some large errors bars on our graphs) and therefore the lack of significant difference observed in the metrics measured may be due to Type II errors due to low sample sizes. Additional variability will have resulted from lack of dietary control. Since the animals were sampled from the wild, we had no knowledge of when they had their last intake of food, or what that comprised.

The Antarctic marine environment is highly seasonal with regard to light and food supply. Although the quality and availability of food oscillates for both primary and secondary consumers, these oscillations are more extreme for primary consumers reliant on the summer phytoplankton bloom [13]. Primary consumers such as suspension feeders experience intense peaks of food supplies during the spring and summer seasons after sea ice melt, mixing of the water column and the phytoplankton bloom [15, 80]. By the end of the late summer period food supplies are ordinarily insufficient to sustain continuous feeding in many suspension feeding species [3, 16]. Hence, we expected to identify lower bacterial diversity in the winter microbiomes of primary consumers compared with other feeding guilds (omnivores/predators).

Our data support this hypothesis as alpha diversity measures were lowest amongst the suspension feeding and carnivore species and highest in the omnivores, *O. victoriae* and *S. neumayeri* (Fig. 1A-B) during winter. Previous feeding studies of Antarctic benthic marine invertebrates (including both *H. steineni* and *C. georgiana* included in this study) found that many invertebrates stop feeding for up to several months during the winter [3, 16, 27]. Winter feeding behaviour is highly variable between species and cessation in feeding does not correlate absolutely with the diminution of the phytoplankton bloom, or indeed extend over the whole winter period, with some species thought to be efficient at utilising the low concentration of nanoplankton which exist in the water column for much of the year [3, 17]. Although previous studies on *E. charcoti* have not investigated feeding biology, the low bacterial diversity identified here in winter suggest it experiences seasonal reductions or cessations in feeding, as seen in the other members of the order dendrochirotida in this study, *C. georgiana* and *H. steineni* [16]. This variable feeding behaviour is not restricted to Antarctic holothurians having also been identified in other species of sea cucumber, where lower alpha diversity metrics have been shown during periods of reduced feeding or aestivation [38, 91]. The differences in gut microbial taxonomic composition between the three Antarctic sea cucumbers are likely due to nuances in feeding with regard to particle size preference. They may also suggest that *E. charcoti* can feed at low levels if some food is available in June, as seen in *C. georgiana* or that it feeds on smaller size classes of food particles that are available over a longer period [3, 17].

It was interesting to note that the scavenger/predator, *O. validus* displayed diversity values comparable to the suspension feeders. Previous studies have noted that despite the flexible feeding strategy of *O. validus* including carnivory, feeding behaviour was still highly seasonal, showing a decrease in winter [74]. A previous analysis of a range of Antarctic secondary consumers found that members of the predator/scavenger feeding guild were similarly affected by environmental seasonality as benthic “herbivores”, with food availability more variable for the former group than anticipated [59]. Although detritivores/omnivores are to a certain extent reliant on the seasonal pulse of phytodetritus, they may be buffered from the seasonal variability of the water column by a persistent long-term sediment food bank [72]. This may explain why the omnivores *S. neumayeri* and *O. victoriae* generally displayed higher bacterial diversity in in winter compared to the other species evaluated. Of the two omnivorous species, *S. neumayeri* displayed wider variation than *O. victoriae*, which could be on account of its cessation in feeding and grazing mechanism of omnivory [6]. The higher bacterial diversity in *O. victoriae* may reflect this species’ wider nutritional and metabolic flexibility over the annual cycle compared with *S*,* neumayeri* [59]. These data could reflect previous suggestions that herbivory was generally associated with higher bacterial diversity than carnivory, on account of the need for bacteria that degrade complex products such as cellulose [92]. Hence, the comparatively low bacterial diversity in the suspension feeders on account of aestivation or reduced feeding, may align with the lower bacterial diversity associated with the more carnivorous lifestyle of the sea star during winter.

Across all six echinoderms, *Pseudomonadota* and *Bacteroidota* were dominant members of the microbiome, aligning with previous investigations on the gut microbiomes of cold water ophiuroids, where *Pseudomonadota* were the most prevalent bacteria representatives [22]. High levels of *Alphaproteobacteria* and *Bacteroidia* contributed to each of these phyla, respectively (Fig. 2B). *Pseudomonadota* and *Bacteroidota* have been observed as dominant members of the surface waters in the Antarctic and sub-Antarctic [1, 73]. However, unlike the observations in this study, these studies found higher levels of SAR 11 clade (*Pelagibacterales*) and *Nitrospinaceae* in winter water samplings from the same bay in Rothera where the animals in this experiment were collected from [73]. These findings offer initial evidence that bacterial communities in the gut microbiomes of these six echinoderms studied here do not directly reflect those of the surrounding seawater, with the animal gut environments exerting active and host-mediated selection of any ingested bacteria [70].

Although we examined the gut microbiota of three different holothurians, as previously noted there were taxonomic differences between them, both in taxonomic composition and correlation (Figs. 1 and 2). *H. steineni* displayed a significant enrichment of *Bacteroidota* in compared to the other two sea cucumbers, *C. georgiana* and *E. charcoti*. The dominance of *Bacteroidota* has been positively correlated with periods of metabolic depression in mammals, due to the ability of bacterial species in this phylum to utilise host-specific gut secretions rather than diet-derived substrates for metabolism [31]. Previous physiological studies of *H. steineni* showed that this species alters protein and nitrogen metabolism across the year, as the diet switches from a mix of lipids, carbohydrates and proteins derived from phytoplankton [86] to using endogenous energy reserves, which are mainly protein, in the food scarce winter [27]. Similarly, a later feeding study of Antarctic marine secondary consumers showed switching of metabolic substrates across the seasons [59]. Hence a similar metabolic switch is likely occurring in these holothurians, with concomitant changes in microbiomes. *Bacteroidota* are important in the degradation of organic matter in natural environments and can degrade dietary polymers such as compounds from plant cell wall material (e.g. cellulose, pectin and xylan) and host-derived carbohydrates which arise from gut secretions (e.g. N-glycans in mucins and chondroitin sulfates) [78]. Therefore, such enzymes are likely to be important for digesting phytoplankton in summer and potentially complex host-substrates in periods of dormancy.

Network reconstruction of the omnivores showed more connectivity than the suspension feeders, potentially reflecting more complex diets and year-round feeding. In these two species there was a microbial cluster of *Bacteroidota*, *Pseudomonadota* and *Actinomycetota* amongst the omnivores in which the genus *Patiriisocius* (order *Flavobacteriales*) formed a central hub. The *Flavobacteriales* are abundant in the Antarctic marine environment, playing a key role in the degradation of algal organic matter through the secretion of extracellular enzymes [85]. The most abundant node in the omnivores’ network was an unclassified member of the family *Anaplasmataceae*, which are intracellular bacteria infecting a diverse range of hosts including wild and domestic animals [33]. *Flavobacteriales* and *Anaplasmataceae* may reflect the omnivore diet including grazing on both phytoplankton and faecal detritus in the benthos.

In contrast, no co-occurrence network was produced for the scavenger/predator *O. validus.* Alpha diversity data showed *Rhodobacteriales* were ubiquitous in *O. validus*. A previous microbiome analysis of *O. validus* among sites in the Antarctic also revealed inter-animal differences in taxonomic composition and high relative abundances of *Rhodobacteraceae* [8]. In this study *Rhodobacteraceae* were coupled with high variations in *Campylobacterota* (Fig. 1 and Supplementary Data Figure S5). *Campylobacterota* have previously been associated with dietary preferences of marine species [5, 10]. *Campylobacterota* have been reported in the coelomic fluid of some echinoderm species, including the commercial sea cucumber *A. japonicus* and two coastal starfish, *Asterias amurensis* and *Patiria pectinifera* [23, 54], but the reason behind this is as yet, not understood, but there may be nutritional benefits for echinoderms for this symbiotic relationship based on diet.

The dietary-specific results from the winter microbiome study were extended to examine seasonal variation in three species obtained in the late summer and winter in 2020 and early summer in 2021. These species represented the different feeding guilds of *H. steineni* (suspension feeder), *O. validus* (scavenger/predator), and *O. victoriae* (omnivore). The effects of seasonality on the microbiomes of these species corroborated findings from the winter microbiome study, with distinct patterns of diversity matching the feeding ecology of the three echinoderms.

The data also reflected the seasonal nature of feeding even in scavenger/predators as identified in previous studies [59]. We expected that there would be higher alpha diversity in this feeding guild due to their broader diets, but in fact diversity was low and seasonally stable, indicating that a broad diet may buffer seasonal change rather than increase diversity. Overall, *O. validus* harboured some unique taxa (*Campylobacterota*) and had a distinct microbial composition with a seasonal effect identified on observed ASVs, but not Shannon diversity. No *Campylobacterota* were identified in *O. validus* during the late summer period, but members of this phylum were present in individuals of *O. validus* in early summer and winter. Abundances were highly variable and could be attributed to feeding status, as these animals were sampled directly from the wild and inter-animal variation.

As regards the other two species studied, there was a trend of increasing bacterial diversity in *H. steineni* towards early summer as the phytoplankton bloom increased (and with it the variety of different sized food items) (Fig. 3A-B). The lower diversity, as measured by observed ASVs, which was likely on account of insufficient food supplies in winter and a cessation in feeding due to the lack of phytoplankton in the water [15, 16]. In contrast the results for *O. victoriae* demonstrated highest microbial gut diversity in winter, where this species would likely have benefitted from the pulse of phytodetritus at the end of summer and faecal detritus from summer visiting megafauna in the sediment as discussed previously. Despite diverging diversity trends in *O. victoriae* and *H. steineni*, both species had increasing relative abundances of *Bacteroidota* towards the winter (Fig. 3C). In contrast, relative abundances of *Bacillota* were highest in both of these two species during early summer. These findings are supported by previous studies. Analysis of the gut microbiota of the sea cucumber, *Stichopus japonicus* from the Yellow Sea, showed an increase of *Bacillota* in spring compared to autumn, whilst *Bacteroidota* decreased in spring compared to the autumn [25]. Distinct increases in *Bacteroidota* were also identified in *A. japonicus* sampled during the minimum productive season compared to the peak productive season at different sites around the coast of Korea [76]. Taxonomic composition could also be related to diatom assemblages and their nutritional composition and whether these were taken in the water column or as phytodetritus. Different species of diatom are known to dominate from early summer to late summer, and the emergence and succession of diatom assemblages driven by water column stratification and sea ice coverage during the preceding winter [68, 80].

Although only a few ASVs overlapped between seasons and species (Supplementary Data Figure S7, Supplementary Table S13), this may partly be attributed to high variability amongst replicates and inter-animal differences. In addition, amongst the few overlapping ASVs it is interesting to note that the marine *Roseobacter* was shared between the omnivores during winter, the echinoderms during the seasonal microbiomes, and the suspension feeders during the warming experiment. The *Roseobacter* lineage contains heterotrophs and aerobic anoxygenic phototrophs that have been associated with algal bloom degradation [84]. This bacterial group were dominant in the microbiomes of the sea star *O. validus* and hypothesised to be preferentially selected by the host from the environment, although its host-specific function is to be investigated further [8]. Inter-animal differences in Bray-Curtis dissimilarities of *H. steineni* and *O. validus* were smallest in late summer, and stable across seasons for *O. victoriae* (Supplementary Data Figure S6). The functional significance of intraspecific variation in invertebrate gut microbiomes is not fully understood, but could be driven by the physiological state of the host, feeding status, composition of the food eaten, the section of the gut sampled and the length of the gut [24] It is to be noted that the digestive tracts of *H. steineni* and *O. validus* differ in length and structure, presenting discrete compartmentalised sections, whilst *O. victoriae* has the simplest, least discrete digestive tract. Different compartments can carry distinctive oxygen, nutrient and pH levels that alter the taxonomic and metabolic profile of microorganisms, and this has been shown to be the case in the gut microbiome of tropical sea cucumbers [66]. Due to the nature and handling of the samples used in this study (i.e. freezing of samples in the Antarctic and then shipment frozen to the UK), it was not possible to confidently distinguish sections of the digestive tract. Future studies investigating bacterial groups associated with specific regions of the digestive tract may provide more detail on seasonal shifts in gut bacterial composition and their functional significance on animal physiology [66]. Although, it was not possible to confidently ascertain the functional significances of taxonomic changes observed in 16S amplicon surveys, due to lack of species resolution and functional redundancies of bacteria from different taxa, seasonal co-occurrence analysis displayed the intriguing emergence of a network cluster of obligate anaerobes from the *Bacillota* phyla which included the *Oscillibacter* genus and *Lachnospiraceae* family, known to be gut bacteria, and identified in the intestinal microbiomes of Antarctic seals and Krill [52, 56].

In a final experiment, we explored the impact of long-term + 2 °C warming on the gut microbiome of the three sea cucumbers. This temperature was chosen on the basis of future IPCC predictions for the region [51]. The sampling and acclimation of these suspension feeders coincided with their period of cessation of feeding during the winter. Therefore, this experiment effectively samples the combined effects of starvation and warming. However, because starvation removes the effect of feeding and different food types, the result is an analysis of the background microbial community and a like for like comparison of the resultant temperature effect. Long-term starvation is not a problem for Antarctic marine invertebrates as due to their incredibly low metabolic rates, they can survive years without food [32]. No impact of + 2 ˚C warming was observed on bacterial diversity of *E. charcoti* and *H. steineni*, after the six-month warming experiment (Fig. 5A-B) and a decrease in observed ASVs, but not Shannon diversity was noted in *C. georgiana* that had been subjected to warming conditions. The former observations reinforce results from previous work showing that prokaryotic communities on settlement panels set at + 2 ˚C above ambient, in Ryder Bay were highly plastic with regard to their temperature tolerances despite macroinvertebrates in the same treatments being highly vulnerable [11]. In addition, culturing of bacteria from the same echinoderms [34] over wide temperature ranges revealed that they are psychrotrophic able to grow above 22 ˚C (Supplementary Data Table S14). Database searches using the ASV data facilitated identification of 10 of these culturable bacteria in this dataset, confirming that thermally resilient bacteria were present within Antarctic echinoderm guts. Although, representation of these cultured representatives was low, higher thermal tolerances of bacteria in Antarctic seawater have been previously recorded [2, 35]. Given these previous data, it was perhaps not surprising to find that + 2 °C of warming had little effect on the community composition of the sea cucumber gut communities.

Even though small incremental changes in temperature may not directly impact bacterial community composition, there are potentially effects at the transcriptional and translational level which may change bacterial protein and metabolite production with consequential effects on the animal host in the longer term resulting in dysbiosis and increased sensitivity to the altered environmental conditions [26]. Such a situation was previously demonstrated in the subtropical urchin, *Lytechinus variegatus*, where marginal changes in bacterial diversity were correlated with pronounced changes in predicted metabolic function [7].

Antarctic marine invertebrates are extremely stenothermal as demonstrated by thermal ramping experiments [62]. Longer-term limits are much lower than those reported from short term acute experiments [62, 63]. It was interesting to note that in thermal resilience trials on all three sea cucumber host species, *E. charcoti* was the least resilient at slow rates of warming, with *C. georgiana* and *H. steineni* showing higher levels of resilience (Supplementary Data, Figure S12), yet it was the *C. georgiana* microbiome community that showed the biggest shift at + 2 °C. The increase in relative abundance of the aerobic chemo-heterotroph *Amaricoccus* in *C. georgiana* and *H. steineni* [47] warrants further investigation as to their potential to buffer warming effects, although their metabolic products (and how they change with temperature) cannot be determined from this experiment. Although the network analysis of all three sea cucumber species illustrated a shift in associations due to patterns of differential abundance at the two temperatures, in *H.steineni* there persisted two clusters based around *Amaricoccus* and *Ilumatobacter*, the latter of which are ubiquitous components of the marine environment [89]. Why there was an apparent separation of clusters in this host species has yet to be determined, but may be due to variable gut sampling and microbial partitioning as mentioned previously. With regard to identifying chronic changes in gut microbiomes associated with environmental change, there are particular challenges in Antarctic species as many host invertebrate species can take up to 9 months to acclimate to even + 2 °C temperature increase [53, 75]. However, although long term experiments are more labour intensive, they are likely to provide more comprehensive answers to the chronic effects of temperature on the gut microbiomes of Antarctic species and potential changes in the food web.

## Conclusions

This study demonstrates that Antarctic echinoderm gut microbiomes are strongly shaped by species, feeding strategy and seasonality and are potentially robust to moderate warming. As expected, lower levels of diversity were associated with periods of reduced productivity and potential aestivation in both primary consumers and some secondary consumers. More pronounced seasonal effects were observed in the omnivorous brittle star, *O. victoriae*, and the observed differences in bacterial diversity may be due to the dependence of its flexible feeding ecology associated with sedimentation. Climate warming in the WAP will impact phenologies, especially the timing of the phytoplankton blooms and sedimentation rates, affecting diet and length of feeding periods which should impact gut microbiomes. Future work should increase sample sizes for greater statistical power and leverage multi-omics techniques to investigate the functional significance of taxonomic changes observed in this study and the mechanisms by which they support in host fitness [40]. The need for multi-omics investigations in warming experiments is particularly emphasised because the absence of taxonomic shifts in the microbiome at the phylum level does not negate the possibility of important species-specific or more global metabolic changes in the microbiota.

## Supplementary Information

Supplementary Material 1.

## Data Availability

16S rRNA amplicon sequence data have been deposited to NCBI Sequence Read Archive (SRA) with BioProject ID: PRJNA1332549.
