# Supplementary material for: The effects of feeding guild, seasonality, and warming on the gut microbiomes of Antarctic echinoderms
Source: BMC Microbiol. 2026 May 12;26:596. doi: 10.1186/s12866-026-05114-4 (PMC13340409; doi:10.1186/s12866-026-05114-4)

**Supplementary Data**

**The effects of feeding guild, seasonality, and warming on the gut microbiomes of Antarctic echinoderms.**

Kudzai Hwengwere^1,2,3^, Benjamin H. Gregson^4^, Susannah J. Salter^5^, Emma Bolton^6^, Lama Alqahtani^7,8^, [Sylvia Rofael](https://www.microbiologyresearch.org/search?value1=Sylvia+Rofael&option1=author&noRedirect=true&sortField=prism_publicationDate&sortDescending=true)^9,10^, Vitor H. Teixeira^7^, Timothy D. McHugh^9^, Grant G. January^11^, Lloyd S. Peck^1^, Mathew Upton^11^, Melody S. Clark^1*^

^1^British Antarctic Survey, Natural Environment Research Council, High Cross, Madingley Road, Cambridge CB3 0ET, UK.

^2^Marine Biology and Ecology Research Centre, School of Biological and Marine Sciences, University of Plymouth, Drake Circus, Plymouth PL4 8AA, UK.

^3^Current address: MRC, Laboratory of Medical Sciences, Imperial College Hammersmith Campus, Du Cane Road, London W12 0HS, UK.

^4^School of Life Sciences, Faculty of Science and Engineering, Anglia Ruskin University, East Road Cambridge, CB1 1PT, UK.

^5^Department of Veterinary Medicine, University of Cambridge, Cambridge CB3 0ES, UK.

^6^Basecamp Research Ltd, Unit 510 Clerkenwell Workshops, 27 Clerkenwell Close, London EC1R 0AT, UK.

^7^UCL Respiratory, Division of Medicine, University College London, London, UK.

^8^Bioengineering institute, Health Sector, King Abdulaziz City for Science and Technology.

Riyadh 11442, Saudi Arabia

^9^UCL, Centre for Clinical Microbiology, Royal Free Campus, London NW3 2QG, UK.

^10^ Faculty of Pharmacy, Alexandria University, Egypt.

^11^School of Biomedical Sciences, University of Plymouth, Derriford Research Facility, Drake Circus, Plymouth PL4 8AA, UK.

**Supplementary Tables**

**Table S1: Water intake temperatures into the Rothera flow through aquarium system.** Water intake is from 10m depth with temperatures recorded via CTD. The 6-month warming experiment was performed at +2°C, tracked in comparison to the water intake temperature

| **Year** | **Month** | **Day** | **Temperature °C at 10m** | **+2°C tracking temperature** |
| --- | --- | --- | --- | --- |
| 2020 | March | 2 | 1.0467 | 3.0467 |
| 2020 | March | 6 | 1.1199 | 3.1199 |
| 2020 | March | 9 | 0.8275 | 2.8275 |
| 2020 | March | 16 | 1.1038 | 3.1038 |
| 2020 | March | 23 | 0.702 | 2.702 |
| 2020 | March | 26 | 0.5624 | 2.5624 |
| 2020 | March | 30 | 0.4802 | 2.4802 |
| 2020 | April | 11 | 0.0217 | 2.0217 |
| 2020 | April | 16 | -0.6027 | 1.3973 |
| 2020 | April | 27 | -0.6343 | 1.3657 |
| 2020 | May | 8 | -0.8796 | 1.1204 |
| 2020 | May | 16 | -1.4578 | 0.5422 |
| 2020 | May | 27 | -0.9166 | 1.0834 |
| 2020 | June | 2 | -1.1694 | 0.8306 |
| 2020 | July | 29 | -1.7795 | 0.2205 |
| 2020 | August | 7 | -1.5868 | 0.4132 |
| 2020 | August | 25 | -1.5517 | 0.4483 |

**Table S2: Packages used in R.4.4.1 and RStudio 2024.04.2+764.**

| Package | Version | Usage |
| --- | --- | --- |
| ggplot2  ggVennDiagram  tidyverse  xlsx  RColorBrewer  patchwork | 3.5.1  1.5.2  2.0.0  0.6.5  1.1.3  1.3.0.9000 | Plotting |
| metagMisc | 0.5.0 | CSS Normalisation |
| phyloseq  microbiome | 1.48.0  1.36.0 | Alpha diversity and Beta diversity and relative abundance |
| vegan  tidyverse  pairwiseAdonis | 2.6.8  2.0.0  0.4.1 | Statistics |
| microViz | 0.12.6 | Filtering phyloseq object |

**Table S3: Summary of amplicon sequencing of all six invertebrates (*C. georgiana*, *E. charcoti*, *H. steineni*, *O. validus*, *O. victoriae*, *S. neumayeri*) during winter.**

|  | ***C. georgiana*** | | |
| --- | --- | --- | --- |
| **Number of reads** | **1** | **2** | **3** |
| Raw | 2158758 | 1635542 | 1304460 |
| Assembled and filtered | 13567 | 27062 | 88111 |
| Normalised | 1150 | 4112 | 3243 |
|  |  |  |  |
|  | ***E. charcoti*** | | |
|  | **1** | **2** | **3** |
| Raw | 693346 | 750840 | 477314 |
| Assembled and filtered | 49732 | 40094 | 5043 |
| Normalised | 1571 | 1122 | 1198 |
|  |  |  |  |
|  | ***H. steineni*** | | |
|  | **1** | **2** | **3** |
| Raw | 5535504 | 6206180 | 3185902 |
| Assembled and filtered | 692605 | 45664 | 169172 |
| Normalised | 60568 | 1318 | 6863 |
|  |  |  |  |
|  | ***O. validus*** | | |
|  | **1** | **2** | **3** |
| Raw | 536832 | 1309700 | 1055494 |
| Assembled and filtered | 2979 | 175340 | 33233 |
| Normalised | 1267 | 23814 | 2230 |
|  |  |  |  |
|  | ***O. victoriae*** | | |
|  | **1** | **2** | **3** |
| Raw | 4762032 | 3843344 | 5889690 |
| Assembled and filtered | 162288 | 19259 | 243838 |
| Normalised | 1121 | 1116 | 1682 |
|  |  |  |  |
|  | ***S***  ***- neumayeri*** | | |
|  | **1** | **2** | **3** |
| Raw | 3812206 | 5136146 | 3655156 |
| Assembled and filtered | 169933 | 229779 | 117319 |
| Normalised | 4907 | 2402 | 1874 |

**Table S4: Summary of amplicon sequencing of *H. steineni*, *O. validus*, and *O. victoriae* in later summer, winter, and early summer.**

|  | ***H. steineni* - late summer** | | | ***H. steineni*  - winter** | | | ***H. steineni* - early summer** | | |
| --- | --- | --- | --- | --- | --- | --- | --- | --- | --- |
| **Number of reads** | **1** | **2** | **1** | **1** | **1** | **2** | **1** | **2** | **3** |
| Raw | 1554942 | 1240666 | 1435500 | 5535504 | 6206180 | 3185902 | 4282906 | 3789702 | 3346122 |
| Assembled and filtered | 52746 | 7809 | 52938 | 692605 | 45664 | 169172 | 20894 | 46724 | 2145 |
| Normalised | 12516 | 8089 | 16386 | 327161 | 36043 | 53344 | 11626 | 9754 | 15105 |
|  |  |  |  |  |  |  |  |  |  |
|  | ***O. validus* - late summer** | | | ***O. validus* - winter** | | | ***O. validus* - early summer** | | |
| **Number of reads** | **1** | **2** | **1** | **1** | **1** | **2** | **1** | **2** | **3** |
| Raw | 790504 | 643574 | 2516940 | 536832 | 1309700 | 1055494 | 6298052 | 4852372 | 6337804 |
| Assembled and filtered | 7072 | 7360 | 145494 | 2979 | 175340 | 33233 | 34210 | 584325 | 176812 |
| Normalised | 32001 | 216473 | 491536 | 35047 | 149861 | 17797 | 14115 | 230781 | 130964 |
|  |  |  |  |  |  |  |  |  |  |
|  | ***O. victoriae* - late summer** | | | ***O. victoriae* – winter** | | | ***O. victoriae* - early summer** | | |
| **Number of reads** | **1** | **2** | **1** | **1** | **1** | **2** | **1** | **2** | **3** |
| Raw | 426136 | 1169258 | 678122 | 4762032 | 3843344 | 5889690 | 4557336 | 1592798 | 2268318 |
| Assembled and filtered | 12651 | 51413 | 27575 | 162288 | 19259 | 243838 | 195118 | 249198 | 93943 |
| Normalised | 5685 | 8401 | 8118 | 9287 | 17229 | 18396 | 257413 | 73100 | 120440 |

**Table S5: Summary of amplicon sequencing of *C. georgiana*, *E. charcoti*, and *H. steineni* during the acclimation experiment.**

|  | ***C. georgiana* - Control** | | | ***C. georgiana* - +2 ˚C** | | |
| --- | --- | --- | --- | --- | --- | --- |
| **Number of reads** | **1** | **2** | **3** | **1** | **2** | **3** |
| Raw | 1226662 | 5307456 | 6098748 | 5837046 | 1241102 | 1581018 |
| Assembled and filtered | 45318 | 207230 | 206961 | 151439 | 29752 | 35268 |
| Normalised | 31783 | 25352 | 41664 | 78285 | 13678 | 9234 |
|  |  |  |  |  |  |  |
|  |  |  |  |  |  |  |
|  | ***E. charcoti* - Control** | | | ***E. charcoti* - +2 ˚C** | | |
| **Number of reads** | **1** | **2** | **3** | **1** | **2** | **3** |
| Raw | 515336 | 1978616 | 516840 | 4697196 | 1203412 | 6698904 |
| Assembled and filtered | 17330 | 75558 | 18873 | 279651 | 77534 | 100482 |
| Normalised | 6914 | 6702 | 5768 | 634132 | 6579 | 28850 |
|  |  |  |  |  |  |  |
|  |  |  |  |  |  |  |
|  | ***H. steineni* - Control** | | | ***H. steineni* - +2 ˚C** | | |
| **Number of reads** | **1** | **2** | **3** | **1** | **2** | **3** |
| Raw | 4708372 | 7267474 | 586716 | 3650758 | 3119672 | 3760176 |
| Assembled and filtered | 11641 | 172786 | 19720 | 25253 | 31070 | 25148 |
| Normalised | 19175 | 24137 | 505644 | 33537 | 19486 | 53507 |

**Table S6: List of ASVs identified as potential contaminants using the Decontam ‘frequency’ method.**

| **ASV** | **Class** | **Order** | **Family** | **Genus** |
| --- | --- | --- | --- | --- |
| ASV_1 | Alphaproteobacteria | Rickettsiales | Rickettsiaceae | #N/A |
| ASV_100 | Actinobacteria | Micrococcales | Micrococcaceae | *Kocuria* |
| ASV_1001 | Acidimicrobiia | Microtrichales | Microtrichaceae | Sva0996 marine group |
| ASV_1009 | Planctomycetes | Planctomycetales | Gimesiaceae | #N/A |
| ASV_101 | Actinobacteria | Micrococcales | Micrococcaceae | *Kocuria* |
| ASV_1012 | Bacteroidia | Flavobacteriales | Flavobacteriaceae | *Olleya* |
| ASV_1014 | Bacteroidia | Cytophagales | Cyclobacteriaceae | *Roseivirga* |
| ASV_1029 | Alphaproteobacteria | Rhodobacterales | Paracoccaceae | *Planktomarina* |
| ASV_103 | Alphaproteobacteria | Hyphomicrobiales | Rhizobiaceae | #N/A |
| ASV_104 | Alphaproteobacteria | Rhodobacterales | Paracoccaceae | #N/A |
| ASV_105 | Bacteroidia | Flavobacteriales | Flavobacteriaceae | *Aureibaculum* |
| ASV_106 | Actinobacteria | Mycobacteriales | Nocardiaceae | *Rhodococcus* |
| ASV_107 | Alphaproteobacteria | Hyphomicrobiales | Rhizobiaceae | #N/A |
| ASV_1079 | Bdellovibrionia | Bdellovibrionales | Pseudobdellovibrionaceae | #N/A |
| ASV_1095 | Bacteroidia | Flavobacteriales | Flavobacteriaceae | *Flavirhabdus* |
| ASV_1096 | Alphaproteobacteria | Kordiimonadales | #N/A | #N/A |
| ASV_11 | Alphaproteobacteria | Rhodobacterales | Paracoccaceae | *Halocynthiibacter* |
| ASV_110 | Alphaproteobacteria | Rickettsiales | Rickettsiaceae | *Candidatus Megaira* |
| ASV_111 | Bacteroidia | Flavobacteriales | Flavobacteriaceae | *Psychroserpens* |
| ASV_1113 | Alphaproteobacteria | Rickettsiales | Rickettsiaceae | #N/A |
| ASV_112 | Alphaproteobacteria | Hyphomicrobiales | Hyphomicrobiaceae | *Filomicrobium* |
| ASV_114 | Alphaproteobacteria | Hyphomicrobiales | Devosiaceae | #N/A |
| ASV_1145 | Alphaproteobacteria | Rhodobacterales | Paracoccaceae | *Parasedimentitalea* |
| ASV_116 | Planctomycetes | Pirellulales | Pirellulaceae | *Mariniblastus* |
| ASV_117 | Campylobacteria | Campylobacterales | Sulfurovaceae | *Sulfurovum* |
| ASV_1190 | Alphaproteobacteria | Rhodobacterales | Paracoccaceae | *Paracoccus* |
| ASV_1193 | Planctomycetes | Planctomycetales | Rubinisphaeraceae | #N/A |
| ASV_1202 | Planctomycetes | Pirellulales | Pirellulaceae | *Mariniblastus* |
| ASV_1205 | Bacteroidia | Flavobacteriales | Flavobacteriaceae | *Aurantivirga* |
| ASV_1216 | Bacteroidia | Flavobacteriales | Flavobacteriaceae | #N/A |
| ASV_122 | Bacteroidia | Flavobacteriales | Flavobacteriaceae | *Ulvibacter* |
| ASV_1229 | Clostridia | Clostridiales | Clostridiaceae | *Clostridium* |
| ASV_123 | Alphaproteobacteria | Rhodobacterales | Paracoccaceae | *Litoreibacter* |
| ASV_124 | Bacteroidia | Flavobacteriales | Flavobacteriaceae | *Zobellia* |
| ASV_125 | Alphaproteobacteria | Rhodobacterales | Paracoccaceae | *Loktanella* |
| ASV_1258 | Planctomycetes | Planctomycetales | Rubinisphaeraceae | *Fuerstia* |
| ASV_126 | Gammaproteobacteria | Gammaproteobacteria Incertae Sedis | Unknown Family | #N/A |
| ASV_1277 | Planctomycetes | Pirellulales | Pirellulaceae | *Bythopirellula* |
| ASV_128 | Fusobacteriia | Fusobacteriales | Fusobacteriaceae | *Psychrilyobacter* |
| ASV_129 | Alphaproteobacteria | Hyphomicrobiales | Hyphomicrobiaceae | *Filomicrobium* |
| ASV_130 | Bacteroidia | Flavobacteriales | Crocinitomicaceae | *Crocinitomix* |
| ASV_131 | Acidimicrobiia | Microtrichales | Iamiaceae | *Aquihabitans* |
| ASV_132 | Alphaproteobacteria | Sneathiellales | Sneathiellaceae | *Sneathiella* |
| ASV_133 | Alphaproteobacteria | Rhodobacterales | Paracoccaceae | *Sulfitobacter* |
| ASV_1355 | Planctomycetes | Pirellulales | Pirellulaceae | *Mariniblastus* |
| ASV_1367 | Alphaproteobacteria | Caulobacterales | Hyphomonadaceae | *Robiginitomaculum* |
| ASV_137 | OM190 | #N/A | #N/A | #N/A |
| ASV_1398 | vadinHA49 | #N/A | #N/A | #N/A |
| ASV_14 | Alphaproteobacteria | Rickettsiales | Rickettsiaceae | #N/A |
| ASV_1402 | Campylobacteria | Campylobacterales | Sulfurovaceae | *Sulfurovum* |
| ASV_141 | Bacteroidia | Flavobacteriales | Flavobacteriaceae | #N/A |
| ASV_1425 | Clostridia | Clostridiales | Clostridiaceae | *Clostridium* |
| ASV_144 | Alphaproteobacteria | Hyphomicrobiales | Methyloligellaceae | #N/A |
| ASV_145 | Alphaproteobacteria | Sphingomonadales | Sphingomonadaceae | #N/A |
| ASV_1459 | Alphaproteobacteria | Micavibrionales | Micavibrionaceae | #N/A |
| ASV_146 | Alphaproteobacteria | Parvibaculales | Parvibaculaceae | #N/A |
| ASV_147 | Alphaproteobacteria | Rhodobacterales | Paracoccaceae | *Monaibacterium* |
| ASV_1475 | Verrucomicrobiia | Opitutales | Puniceicoccaceae | *Lentimonas* |
| ASV_148 | Alphaproteobacteria | Rhodobacterales | Paracoccaceae | *Sulfitobacter* |
| ASV_149 | Alphaproteobacteria | Hyphomicrobiales | Rhizobiaceae | *Pseudahrensia* |
| ASV_15 | Alphaproteobacteria | Rhodobacterales | Paracoccaceae | #N/A |
| ASV_1513 | Gammaproteobacteria | Tenderiales | Tenderiaceae | *Candidatus* Tenderia |
| ASV_152 | Bacteroidia | Flavobacteriales | Flavobacteriaceae | *Lutimonas* |
| ASV_1541 | OM190 | #N/A | #N/A | #N/A |
| ASV_1546 | Bacteroidia | Flavobacteriales | Flavobacteriaceae | *Gilvibacter* |
| ASV_155 | Bacteroidia | Flavobacteriales | Flavobacteriaceae | *Winogradskyella* |
| ASV_157 | Actinobacteria | Mycobacteriales | Tsukamurellaceae | *Tsukamurella* |
| ASV_1574 | Phycisphaerae | Phycisphaerales | Phycisphaeraceae | SM1A02 |
| ASV_161 | Alphaproteobacteria | Hyphomicrobiales | Hyphomicrobiaceae | *Filomicrobium* |
| ASV_1615 | Acidimicrobiia | Microtrichales | Microtrichaceae | IMCC26207 |
| ASV_1619 | Alphaproteobacteria | Rickettsiales | Mitochondria | #N/A |
| ASV_163 | Alphaproteobacteria | Hyphomicrobiales | Rhizobiaceae | *Pseudahrensia* |
| ASV_165 | Alphaproteobacteria | Rickettsiales | Mitochondria | #N/A |
| ASV_167 | Alphaproteobacteria | Rhodobacterales | Paracoccaceae | *Sulfitobacter* |
| ASV_168 | Planctomycetes | Pirellulales | Pirellulaceae | *Rubripirellula* |
| ASV_1686 | Planctomycetes | Pirellulales | Pirellulaceae | *Bythopirellula* |
| ASV_17 | Alphaproteobacteria | Hyphomicrobiales | Hyphomicrobiaceae | *Filomicrobium* |
| ASV_1708 | Alphaproteobacteria | Rickettsiales | AB1 | #N/A |
| ASV_171 | Alphaproteobacteria | Rickettsiales | Rickettsiaceae | *Candidatus* Megaira |
| ASV_172 | Bacteroidia | Flavobacteriales | Flavobacteriaceae | *Lutimonas* |
| ASV_173 | Actinobacteria | Mycobacteriales | Corynebacteriaceae | *Corynebacterium* |
| ASV_1730 | Bacteroidia | Flavobacteriales | Flavobacteriaceae | *Gillisia* |
| ASV_1738 | Planctomycetes | Planctomycetales | Gimesiaceae | #N/A |
| ASV_174 | Actinobacteria | Micrococcales | Microbacteriaceae | *Microbacterium* |
| ASV_1749 | Actinobacteria | Mycobacteriales | Nocardiaceae | *Rhodococcus* |
| ASV_176 | Bacteroidia | Flavobacteriales | Flavobacteriaceae | *Lutimonas* |
| ASV_1761 | vadinHA49 | #N/A | #N/A | #N/A |
| ASV_177 | Alphaproteobacteria | Pelagibacterales | Clade I | Clade Ia |
| ASV_1792 | Bacteroidia | Flavobacteriales | Schleiferiaceae | #N/A |
| ASV_1809 | Planctomycetes | Pirellulales | Pirellulaceae | *Bythopirellula* |
| ASV_181 | Bacteroidia | Flavobacteriales | Flavobacteriaceae | *Aureibaculum* |
| ASV_182 | Bacteroidia | Flavobacteriales | Flavobacteriaceae | *Lutibacter* |
| ASV_1848 | Alphaproteobacteria | Rickettsiales | Mitochondria | #N/A |
| ASV_186 | Alphaproteobacteria | Rhodobacterales | Paracoccaceae | #N/A |
| ASV_1891 | Gammaproteobacteria | #N/A | #N/A | #N/A |
| ASV_1893 | Gracilibacteria | JGI 0000069-P22 | #N/A | #N/A |
| ASV_1897 | Planctomycetes | Planctomycetales | Gimesiaceae | #N/A |
| ASV_19 | Alphaproteobacteria | Rhodobacterales | Paracoccaceae | *Sulfitobacter* |
| ASV_190 | Acidimicrobiia | Microtrichales | Iamiaceae | *Aquihabitans* |
| ASV_1938 | Clostridia | Peptostreptococcales-Tissierellales | Peptostreptococcaceae | *Romboutsia* |
| ASV_194 | Bacteroidia | Flavobacteriales | Flavobacteriaceae | #N/A |
| ASV_199 | Clostridia | Clostridiales | Clostridiaceae | *Clostridium* |
| ASV_20 | Alphaproteobacteria | Rhodobacterales | Paracoccaceae | *Roseobacter* |
| ASV_200 | Bacteroidia | Flavobacteriales | Flavobacteriaceae | #N/A |
| ASV_201 | Alphaproteobacteria | Hyphomicrobiales | Hyphomicrobiaceae | *Filomicrobium* |
| ASV_2014 | Bacteroidia | Flavobacteriales | Flavobacteriaceae | *Formosa* |
| ASV_2019 | Planctomycetes | Pirellulales | Pirellulaceae | *Mariniblastus* |
| ASV_202 | Bacteroidia | Flavobacteriales | Flavobacteriaceae | *Aureibaculum* |
| ASV_2029 | Bdellovibrionia | Bdellovibrionales | Pseudobdellovibrionaceae | #N/A |
| ASV_2030 | Planctomycetes | Pirellulales | Pirellulaceae | *Mariniblastus* |
| ASV_2063 | Planctomycetes | Pirellulales | Pirellulaceae | *Rhodopirellula* |
| ASV_207 | Planctomycetes | Planctomycetales | Gimesiaceae | #N/A |
| ASV_2071 | Alphaproteobacteria | Rhodospirillales | #N/A | #N/A |
| ASV_2081 | Bacteroidia | Flavobacteriales | Flavobacteriaceae | #N/A |
| ASV_2086 | Alphaproteobacteria | Micavibrionales | Micavibrionaceae | #N/A |
| ASV_21 | Clostridia | Clostridiales | Clostridiaceae | *Clostridium* |
| ASV_211 | Bacteroidia | Flavobacteriales | Flavobacteriaceae | *Maribacter* |
| ASV_212 | Alphaproteobacteria | Rhodobacterales | Paracoccaceae | *Albimonas* |
| ASV_213 | Bacteroidia | Flavobacteriales | Cryomorphaceae | *Cryomorpha* |
| ASV_2131 | Bacteroidia | Flavobacteriales | Cryomorphaceae | #N/A |
| ASV_2142 | Bacteroidia | Flavobacteriales | Flavobacteriaceae | *Maribacter* |
| ASV_215 | Verrucomicrobiia | Opitutales | Puniceicoccaceae | *Lentimonas* |
| ASV_22 | Clostridia | Peptostreptococcales-Tissierellales | Peptostreptococcaceae | *Paraclostridium* |
| ASV_221 | Bacteroidia | Flavobacteriales | Flavobacteriaceae | *Lutimonas* |
| ASV_222 | Bacteroidia | Flavobacteriales | Flavobacteriaceae | NS5 marine group |
| ASV_2223 | Acidimicrobiia | Microtrichales | Ilumatobacteraceae | *Ilumatobacter* |
| ASV_225 | Acidimicrobiia | Actinomarinales | #N/A | #N/A |
| ASV_2278 | Alphaproteobacteria | Rickettsiales | Rickettsiaceae | #N/A |
| ASV_2326 | Gammaproteobacteria | Coxiellales | Coxiellaceae | *Coxiella* |
| ASV_235 | Alphaproteobacteria | Rhodobacterales | Paracoccaceae | *Roseobacter* clade NAC11-7 lineage |
| ASV_238 | Planctomycetes | Pirellulales | Pirellulaceae | *Mariniblastus* |
| ASV_2381 | Alphaproteobacteria | Rickettsiales | Mitochondria | #N/A |
| ASV_2391 | Planctomycetes | Pirellulales | Pirellulaceae | *Bremerella* |
| ASV_24 | Alphaproteobacteria | #N/A | #N/A | #N/A |
| ASV_242 | Bacteroidia | Flavobacteriales | Flavobacteriaceae | *Flavirhabdus* |
| ASV_2445 | OM190 | #N/A | #N/A | #N/A |
| ASV_245 | Bacteroidia | Flavobacteriales | Flavobacteriaceae | *Arcticiflavibacter* |
| ASV_2462 | Planctomycetes | Pirellulales | Pirellulaceae | *Bremerella* |
| ASV_2476 | Gammaproteobacteria | Enterobacterales | Moritellaceae | *Moritella* |
| ASV_248 | Alphaproteobacteria | Rhodobacterales | Paracoccaceae | *Amylibacter* |
| ASV_249 | Nitrospiria | Nitrospirales | Nitrospiraceae | *Nitrospira* |
| ASV_25 | Alphaproteobacteria | Rhodobacterales | Paracoccaceae | *Roseobacter* clade NAC11-7 lineage |
| ASV_251 | Alphaproteobacteria | Hyphomicrobiales | Methyloligellaceae | #N/A |
| ASV_252 | Bacteroidia | Flavobacteriales | Flavobacteriaceae | *Aquibacter* |
| ASV_254 | Planctomycetes | Planctomycetales | Gimesiaceae | #N/A |
| ASV_255 | Bacteroidia | Flavobacteriales | Flavobacteriaceae | *Lutimonas* |
| ASV_257 | Acidimicrobiia | Microtrichales | Ilumatobacteraceae | *Ilumatobacter* |
| ASV_26 | Clostridia | Clostridiales | Clostridiaceae | *Clostridium* |
| ASV_261 | Bacteroidia | Flavobacteriales | Flavobacteriaceae | #N/A |
| ASV_262 | Acidimicrobiia | Microtrichales | Ilumatobacteraceae | *Ilumatobacter* |
| ASV_2630 | Thermoleophilia | Solirubrobacterales | 67-14 | #N/A |
| ASV_268 | Planctomycetes | Pirellulales | Pirellulaceae | *Rhodopirellula* |
| ASV_2728 | Bacteroidia | Flavobacteriales | Flavobacteriaceae | *Wenyingzhuangia* |
| ASV_2749 | Gammaproteobacteria | #N/A | #N/A | #N/A |
| ASV_275 | Alphaproteobacteria | Parvibaculales | PS1 clade | #N/A |
| ASV_2781 | Planctomycetes | Planctomycetales | Gimesiaceae | Gimesia |
| ASV_2799 | Alphaproteobacteria | Rickettsiales | Mitochondria | #N/A |
| ASV_28 | Alphaproteobacteria | Sphingomonadales | Sphingomonadaceae | *Sphingobium* |
| ASV_280 | Bacteroidia | Flavobacteriales | Flavobacteriaceae | *Patiriisocius* |
| ASV_2806 | Alphaproteobacteria | Rhodobacterales | Paracoccaceae | *Yoonia* |
| ASV_282 | Bdellovibrionia | Bdellovibrionales | Pseudobdellovibrionaceae | #N/A |
| ASV_283 | Alphaproteobacteria | Rickettsiales | #N/A | #N/A |
| ASV_2844 | Omnitrophia | Omnitrophales | koll11 | #N/A |
| ASV_286 | Actinobacteria | Propionibacteriales | Propionibacteriaceae | *Cutibacterium* |
| ASV_29 | Acidimicrobiia | Microtrichales | Ilumatobacteraceae | *Ilumatobacter* |
| ASV_290 | Actinobacteria | Micrococcales | Micrococcaceae | *Micrococcus* |
| ASV_291 | Planctomycetes | Pirellulales | Pirellulaceae | *Rhodopirellula* |
| ASV_297 | Alphaproteobacteria | Hyphomicrobiales | Rhizobiaceae | *Pseudahrensia* |
| ASV_2994 | Bacteroidia | Flavobacteriales | Flavobacteriaceae | *Ulvibacter* |
| ASV_300 | Actinobacteria | Micrococcales | Microbacteriaceae | *Microbacterium* |
| ASV_302 | Alphaproteobacteria | Rhodobacterales | Paracoccaceae | #N/A |
| ASV_303 | Bacteroidia | Chitinophagales | Chitinophagaceae | #N/A |
| ASV_304 | Bacteroidia | Flavobacteriales | Flavobacteriaceae | #N/A |
| ASV_305 | Bacteroidia | Flavobacteriales | Flavobacteriaceae | *Aureibaculum* |
| ASV_3099 | Alphaproteobacteria | Rhodobacterales | Paracoccaceae | *Sulfitobacter* |
| ASV_310 | Acidimicrobiia | Microtrichales | Ilumatobacteraceae | *Ilumatobacter* |
| ASV_3100 | Gammaproteobacteria | Enterobacterales | Psychromonadaceae | *Psychromonas* |
| ASV_312 | Alphaproteobacteria | Rhodobacterales | Paracoccaceae | *Parasedimentitalea* |
| ASV_3183 | Alphaproteobacteria | Rickettsiales | Rickettsiaceae | #N/A |
| ASV_3202 | Alphaproteobacteria | Rhodobacterales | Paracoccaceae | *Sulfitobacter* |
| ASV_3228 | Lentisphaeria | Lentisphaerales | Lentisphaeraceae | *Lentisphaera* |
| ASV_3229 | Gammaproteobacteria | Enterobacterales | Moritellaceae | #N/A |
| ASV_327 | Bacteroidia | Flavobacteriales | Flavobacteriaceae | *NS5 marine group* |
| ASV_3273 | Gammaproteobacteria | Pseudomonadales | Halieaceae | *Halioglobus* |
| ASV_329 | Planctomycetes | Pirellulales | Pirellulaceae | *Bythopirellula* |
| ASV_3293 | Lentisphaeria | P.palmC41 | #N/A | #N/A |
| ASV_33 | Alphaproteobacteria | Rhodobacterales | Paracoccaceae | *Litoreibacter* |
| ASV_334 | Alphaproteobacteria | Sphingomonadales | Sphingomonadaceae | *Novosphingobium* |
| ASV_336 | Acidimicrobiia | Actinomarinales | #N/A | #N/A |
| ASV_34 | Acidimicrobiia | Microtrichales | Ilumatobacteraceae | *Ilumatobacter* |
| ASV_35 | Actinobacteria | Propionibacteriales | Propionibacteriaceae | *Cutibacterium* |
| ASV_353 | Bacilli | Lactobacillales | Carnobacteriaceae | *Dolosigranulum* |
| ASV_357 | Bacteroidia | Flavobacteriales | Flavobacteriaceae | #N/A |
| ASV_3572 | Gammaproteobacteria | Enterobacterales | Pasteurellaceae | #N/A |
| ASV_36 | Alphaproteobacteria | Hyphomicrobiales | Beijerinckiaceae | *Methylorubrum* |
| ASV_365 | Acidimicrobiia | Microtrichales | Microtrichaceae | #N/A |
| ASV_381 | Bacteroidia | Flavobacteriales | Flavobacteriaceae | #N/A |
| ASV_383 | Planctomycetes | Pirellulales | Pirellulaceae | *Bythopirellula* |
| ASV_387 | Bacteroidia | Flavobacteriales | Flavobacteriaceae | #N/A |
| ASV_392 | Actinobacteria | Micrococcales | Micrococcaceae | *Kocuria* |
| ASV_394 | Nitrospiria | Nitrospirales | Nitrospiraceae | *Nitrospira* |
| ASV_395 | Bacteroidia | Flavobacteriales | Flavobacteriaceae | *Flavirhabdus* |
| ASV_399 | Planctomycetes | Pirellulales | Pirellulaceae | *Bythopirellula* |
| ASV_4 | Alphaproteobacteria | Rhodobacterales | Paracoccaceae | *Sulfitobacter* |
| ASV_409 | Planctomycetes | Pirellulales | Pirellulaceae | *Pir4 lineage* |
| ASV_411 | Bacteroidia | Flavobacteriales | Flavobacteriaceae | #N/A |
| ASV_414 | Bacteroidia | Flavobacteriales | Flavobacteriaceae | *Pibocella* |
| ASV_420 | Planctomycetes | Pirellulales | Pirellulaceae | *Rubripirellula* |
| ASV_424 | Planctomycetes | Pirellulales | Pirellulaceae | *Mariniblastus* |
| ASV_43 | Alphaproteobacteria | Rhodobacterales | Paracoccaceae | *Pelagimonas* |
| ASV_432 | Alphaproteobacteria | Rhodobacterales | Paracoccaceae | *Parasedimentitalea* |
| ASV_4345 | Chlamydiia | Chlamydiales | #N/A | #N/A |
| ASV_44 | Alphaproteobacteria | Caulobacterales | Hyphomonadaceae | *Hellea* |
| ASV_442 | Clostridia | Peptostreptococcales-Tissierellales | Peptostreptococcaceae | *Paraclostridium* |
| ASV_444 | Actinobacteria | Micrococcales | Microbacteriaceae | #N/A |
| ASV_4529 | Gammaproteobacteria | Pseudomonadales | Halieaceae | #N/A |
| ASV_468 | Bacteroidia | Flavobacteriales | Flavobacteriaceae | *Maribacter* |
| ASV_474 | Planctomycetes | Pirellulales | Pirellulaceae | *Mariniblastus* |
| ASV_48 | Alphaproteobacteria | Rhodobacterales | Paracoccaceae | *Roseobacter* clade CHAB-I-5 lineage |
| ASV_480 | Clostridia | Clostridiales | Clostridiaceae | *Clostridium* |
| ASV_4802 | Gammaproteobacteria | Pseudomonadales | Halieaceae | *Marimicrobium* |
| ASV_485 | Alphaproteobacteria | Rickettsiales | Mitochondria | #N/A |
| ASV_4880 | Alphaproteobacteria | Rhodobacterales | Paracoccaceae | *Pelagimonas* |
| ASV_49 | Alphaproteobacteria | Hyphomicrobiales | Rhizobiaceae | #N/A |
| ASV_493 | Alphaproteobacteria | Rhodobacterales | Paracoccaceae | *Parasedimentitalea* |
| ASV_4944 | Gammaproteobacteria | Pseudomonadales | Halieaceae | *Halioglobus* |
| ASV_496 | Actinobacteria | Propionibacteriales | Propionibacteriaceae | #N/A |
| ASV_5 | Alphaproteobacteria | Hyphomicrobiales | Xanthobacteraceae | *Afipia* |
| ASV_52 | Alphaproteobacteria | Hyphomicrobiales | Rhizobiaceae | *Pseudahrensia* |
| ASV_521 | Alphaproteobacteria | Rhodobacterales | Paracoccaceae | *Yoonia* |
| ASV_5262 | Gammaproteobacteria | Pseudomonadales | Halieaceae | *Marimicrobium* |
| ASV_5292 | Gammaproteobacteria | Pseudomonadales | Halieaceae | *Halioglobus* |
| ASV_53 | Bacteroidia | Flavobacteriales | Flavobacteriaceae | *Winogradskyella* |
| ASV_534 | Alphaproteobacteria | Rhodobacterales | Paracoccaceae | *Jannaschia* |
| ASV_5417 | Gammaproteobacteria | #N/A | #N/A | #N/A |
| ASV_542 | Bacteroidia | Flavobacteriales | Flavobacteriaceae | *Polaribacter* |
| ASV_547 | Alphaproteobacteria | Hyphomicrobiales | Rhizobiaceae | #N/A |
| ASV_548 | Anaerolineae | Caldilineales | Caldilineaceae | #N/A |
| ASV_55 | Alphaproteobacteria | Rhodobacterales | Paracoccaceae | *Sulfitobacter* |
| ASV_557 | Campylobacteria | Campylobacterales | Arcobacteraceae | *Arcobacter* |
| ASV_56 | Bacteroidia | Flavobacteriales | Weeksellaceae | *Cloacibacterium* |
| ASV_560 | Bacteroidia | Flavobacteriales | NS9 marine group | #N/A |
| ASV_57 | Bacteroidia | Flavobacteriales | Flavobacteriaceae | *Lutimonas* |
| ASV_572 | Bacteroidia | Flavobacteriales | Flavobacteriaceae | *Ulvibacter* |
| ASV_582 | Alphaproteobacteria | Hyphomicrobiales | Rhizobiaceae | *Pseudahrensia* |
| ASV_6 | Alphaproteobacteria | Rhodobacterales | Paracoccaceae | *Yoonia* |
| ASV_60 | Bacteroidia | Flavobacteriales | Flavobacteriaceae | *Maribacter* |
| ASV_601 | Clostridia | Clostridiales | Clostridiaceae | *Clostridium* |
| ASV_608 | Actinobacteria | Propionibacteriales | Nocardioidaceae | *Nocardioides* |
| ASV_615 | Bacteroidia | Flavobacteriales | Flavobacteriaceae | *Pricia* |
| ASV_62 | Acidimicrobiia | Microtrichales | Ilumatobacteraceae | *Ilumatobacter* |
| ASV_6239 | Planctomycetes | Planctomycetales | Rubinisphaeraceae | *Planctomicrobium* |
| ASV_63 | Acidimicrobiia | Microtrichales | Ilumatobacteraceae | *Ilumatobacter* |
| ASV_64 | Alphaproteobacteria | Rhodobacterales | Paracoccaceae | *Roseobacter* clade NAC11-7 lineage |
| ASV_65 | Bacteroidia | Flavobacteriales | Flavobacteriaceae | *Maritimimonas* |
| ASV_663 | Clostridia | Peptostreptococcales-Tissierellales | Peptostreptococcaceae | #N/A |
| ASV_665 | Bacteroidia | Flavobacteriales | Flavobacteriaceae | #N/A |
| ASV_666 | Planctomycetes | Pirellulales | Pirellulaceae | #N/A |
| ASV_680 | Planctomycetes | Pirellulales | Pirellulaceae | *Rhodopirellula* |
| ASV_70 | Alphaproteobacteria | Rhodobacterales | Paracoccaceae | *Rubellimicrobium* |
| ASV_7077 | Gammaproteobacteria | Pseudomonadales | Gven-F17 | #N/A |
| ASV_71 | Bacteroidia | Flavobacteriales | Flavobacteriaceae | #N/A |
| ASV_723 | Acidimicrobiia | Microtrichales | Ilumatobacteraceae | *Ilumatobacter* |
| ASV_7284 | Gammaproteobacteria | Enterobacterales | Psychromonadaceae | *Psychromonas* |
| ASV_73 | Alphaproteobacteria | Rhodobacterales | Paracoccaceae | *Parasedimentitalea* |
| ASV_733 | Alphaproteobacteria | Rhodobacterales | Paracoccaceae | *Tabrizicola* |
| ASV_745 | OM190 | #N/A | #N/A | #N/A |
| ASV_748 | Clostridia | Peptostreptococcales-Tissierellales | Peptostreptococcaceae | #N/A |
| ASV_754 | Bacteroidia | Flavobacteriales | Flavobacteriaceae | *Aureibaculum* |
| ASV_7567 | Chlamydiia | Chlamydiales | #N/A | #N/A |
| ASV_758 | Planctomycetes | Planctomycetales | Rubinisphaeraceae | #N/A |
| ASV_762 | Planctomycetes | Pirellulales | Pirellulaceae | *Rubripirellula* |
| ASV_766 | Bacteroidia | Flavobacteriales | Flavobacteriaceae | *Aquibacter* |
| ASV_785 | Planctomycetes | Pirellulales | Pirellulaceae | *Mariniblastus* |
| ASV_798 | Alphaproteobacteria | Rhodobacterales | Paracoccaceae | *Yoonia* |
| ASV_80 | Alphaproteobacteria | Rhodobacterales | Paracoccaceae | *Amaricoccus* |
| ASV_800 | Bacteroidia | Bacteroidales | Marinifilaceae | *Ancylomarina* |
| ASV_803 | KD4-96 | #N/A | #N/A | #N/A |
| ASV_81 | Alphaproteobacteria | Rickettsiales | Rickettsiaceae | #N/A |
| ASV_82 | Acidimicrobiia | Microtrichales | Ilumatobacteraceae | *Ilumatobacter* |
| ASV_823 | Bacteroidia | Flavobacteriales | Flavobacteriaceae | *Marixanthomonas* |
| ASV_826 | Actinobacteria | Mycobacteriales | Mycobacteriaceae | *Mycobacterium* |
| ASV_83 | Bacteroidia | Flavobacteriales | Flavobacteriaceae | *Winogradskyella* |
| ASV_84 | Actinobacteria | Mycobacteriales | Nocardiaceae | *Rhodococcus* |
| ASV_8437 | Gammaproteobacteria | Pseudomonadales | Spongiibacteraceae | #N/A |
| ASV_854 | Alphaproteobacteria | Rhodobacterales | Paracoccaceae | *Sulfitobacter* |
| ASV_861 | Bacteroidia | Flavobacteriales | Flavobacteriaceae | *Psychroserpens* |
| ASV_867 | Planctomycetes | Pirellulales | Pirellulaceae | *Bythopirellula* |
| ASV_871 | Bacteroidia | Flavobacteriales | Crocinitomicaceae | *Crocinitomix* |
| ASV_874 | Alphaproteobacteria | Hyphomicrobiales | Rhizobiaceae | *Pseudahrensia* |
| ASV_884 | Bacteroidia | Flavobacteriales | Flavobacteriaceae | *Maribacter* |
| ASV_886 | Clostridia | Clostridiales | Clostridiaceae | *Clostridium* |
| ASV_887 | Alphaproteobacteria | Rhodobacterales | Paracoccaceae | #N/A |
| ASV_89 | Bacteroidia | Flavobacteriales | Flavobacteriaceae | *Pseudofulvibacter* |
| ASV_898 | Alphaproteobacteria | Rhodobacterales | Paracoccaceae | *Amaricoccus* |
| ASV_9 | Bacteroidia | Cytophagales | Cyclobacteriaceae | *Fulvivirga* |
| ASV_91 | Clostridia | Clostridiales | Clostridiaceae | *Clostridium* |
| ASV_911 | Alphaproteobacteria | Sphingomonadales | Sphingomonadaceae | *Sphingobium* |
| ASV_92 | Bacteroidia | Flavobacteriales | Weeksellaceae | *Cloacibacterium* |
| ASV_921 | Alphaproteobacteria | Caulobacterales | Hyphomonadaceae | *Fretibacter* |
| ASV_930 | Actinobacteria | Mycobacteriales | Corynebacteriaceae | *Lawsonella* |
| ASV_933 | Bdellovibrionia | Bdellovibrionales | Pseudobdellovibrionaceae | #N/A |
| ASV_96 | Alphaproteobacteria | Rhodobacterales | Paracoccaceae | *Pelagicola* |
| ASV_962 | Alphaproteobacteria | Rhodobacterales | Paracoccaceae | *Sulfitobacter* |
| ASV_97 | Alphaproteobacteria | Hyphomicrobiales | Methyloligellaceae | *Methyloceanibacter* |
| ASV_980 | OM190 | #N/A | #N/A | #N/A |
| ASV_982 | Alphaproteobacteria | Defluviicoccales | #N/A | #N/A |
| ASV_99 | Bacteroidia | Flavobacteriales | Flavobacteriaceae | *Pseudozobellia* |
| ASV_994 | Planctomycetes | Pirellulales | Pirellulaceae | *Mariniblastus* |

**Table S7: Statistics for alpha diversity results from winter microbiome experiment.**

| **Winter microbiome - Observed ASVs** | | | | |  |
| --- | --- | --- | --- | --- | --- |
| One-Way ANOVA | | d.f. | F | *p* value | Power |
| Model: species | | 5 | 3.104 | 0.050 | 0.492 |
| **Winter microbiome - Shannon** | | | |  |  |
| One-Way ANOVA | | d.f. | F | *p* value | Power |
| Model: species | | 5 | 3.122 | 0.049 | 0.496 |
|  | Comparison | Diff of Means | q | *p value* |  |
|  | *O. victoriae vs H. steineni* | 3.255 | 4.866 | 0.044 |  |
|  | *O. victoriae vs O. validus* | 2.97 | 4.441 | 0.072 |  |
|  | *O. victoriae vs C. georgiana* | 2.112 | 3.158 | 0.292 |  |
|  | *O. victoriae vs E. charcoti* | 1.871 | 2.797 | 0.406 |  |
|  | *O. victoriae vs S. neumayeri* | 1.309 | 1.957 | 0.736 |  |
|  | *S. neumayeri vs H. steineni* | 1.946 | 2.909 | 0.368 |  |
|  | *S. neumayeri vs O. validus* | 1.662 | 2.484 | 0.524 |  |
|  | *S. neumayeri vs C. georgiana* | 0.803 | 1.201 | 0.952 |  |
|  | *S. neumayeri vs E. charcoti* | 0.562 | 0.84 | 0.99 |  |
|  | *E. charcoti vs H. steineni* | 1.384 | 2.069 | 0.692 |  |
|  | *E. charcoti vs O. validus* | 1.099 | 1.644 | 0.846 |  |
|  | *E. charcoti vs C. georgiana* | 0.241 | 0.361 | 1 |  |
|  | *C. georgiana vs H. steineni* | 1.143 | 1.708 | 0.825 |  |
|  | *C. georgiana vs O. validus* | 0.858 | 1.283 | 0.937 |  |
|  | *O. validus vs H. steineni* | 0.284 | 0.425 | 1 |  |

**Table S8 - Statistics for beta diversity results from winter microbiome experiment.**

| **Winter microbiome - Bray-Curtis dissimilarity distances** | | | | |
| --- | --- | --- | --- | --- |
| PERMANOVA | d.f. | *r*^2^ | F | Pr(>F) |
| Model: species | 5 | 0.46684 | 2.101463 | 0.001 |
| **Homogeneity of group dispersions in Bray Curtis dissimilarity distances** | | | | |
| One-Way ANOVA | d.f. | F | Pr(>F) |  |
| Model: species | 5 | 1.9059 | 0.167 |  |

**Table S9 - Statistics for alpha diversity results from seasonal microbiome experiment.**

| **Season microbiome - Observed ASVs** | | | | | |
| --- | --- | --- | --- | --- | --- |
| One-Way ANOVA | *H. steineni* | d.f. | F | *p* value | Power |
| Model: season | | 2 | 1.673 | 0.265 | 0.119 |
| One-Way ANOVA | *O. validus* | d.f. | F | *p* value | Power |
| Model: season | | 2 | 13.746 | 0.006 | 0.936 |
| Post hoc Tukey test | Comparison | Diff of means | q | *p* value |  |
|  | *O. validus*: early summer vs late summer | 165.333 | 7.137 | 0.006 |  |
|  | *O. validus*: early summer vs winter | 123.000 | 5.310 | 0.022 |  |
|  | *O. validus*: winter vs late summer | 42.333 | 1.828 | 0.449 |  |
| One-Way ANOVA | *O. victoriae* | d.f. | F | *p value* | Power |
| Model: season | | 2 | 5.410 | 0.045 | 0.526 |
| Post hoc Tukey test | Comparison | Diff of means | q | *p* value |  |
|  | *O. victoriae*: winter vs late summer | 225.667 | 4.183 | 0.057 |  |
|  | *O. victoriae*: winter vs early summer | 208.000 | 3.855 | 0.077 |  |
|  | *O. victoriae*: early summer vs late summer | 17.667 | 0.327 | 0.971 |  |
| **Season microbiome – Shannon** | | | | | |
| One-Way ANOVA | *H. steineni* | d.f. | F | *p* value | Power |
| Model: season | | 2 | 7.152 | 0.026 | 0.672 |
| Post hoc Tukey test | Comparison | Diff of means | q | *p* value |  |
|  | *H. steineni*: winter vs early summer | 2.372 | 5.046 | 0.028 |  |
|  | *H. steineni*: winter vs late summer | 0.464 | 0.988 | 0.773 |  |
|  | *H. steineni*: late vs early summer | 1.908 | 4.059 | 0.064 |  |
| One-Way ANOVA | *O. validus* | d.f. | F | *p* value | Power |
| Model: season | | 2 | 0.447 | 0.050 | 0.050 |
| One-Way ANOVA | *O. victoriae* | d.f. | F | *p* value | Power |
| Model: season | | 2 | 10.495 | 0.011 | 0.850 |
| Post hoc Tukey test | Comparison | Diff of means | q | *p* value |  |
|  | *O. victoriae*: winter vs early summer | 2.335 | 6.450 | 0.009 |  |
|  | *O. victoriae*: winter vs late summer | 1.003 | 2.783 | 0.201 |  |
|  | *O. victoriae*: late vs early summer | 1.321 | 3.666 | 0.091 |  |

**Table S10 - Statistics for beta diversity results from seasonal microbiome experiment.**

| **Season microbiome - Bray- Curtis dissimilarity score** | | | | |
| --- | --- | --- | --- | --- |
| PERMANOVA | d.f. | *r*^2^ | F | Pr(>F) |
| Model: species | 2 | 0.113175 | 1.531417 | 0.001 |
| Model: season | 2 | 0.091775 | 1.212584 | 0.024 |
| Model: species and season | 8 | 0.399709 | 1.498184 | 0.001 |
| Model: *H. steineni* - season | 2 | 0.340727 | 1.550464 | 0.016 |
| Model: *O. validus -* season | 2 | 0.304723 | 1.314826 | 0.241 |
| Model: *O. victoriae* - season | 2 | 0.513988 | 3.172681 | 0.004 |
| Model: *H. steineni* - season | 2 | 0.340727 | 1.550464 | 0.016 |
| **Homogeneity of group dispersions in Bray Curtis dissimilarity distances** | | | | |
| One-Way ANOVA | d.f. | F | Pr(>F) |  |
| Model: species | 2 | 1.4656 | 0.2509 |  |
| Model: season | 2 | 0.2577 | 0.775 |  |
| Model: *O. validus* - season | 2 | 0.3837 | 0.6969 |  |
| Model: O. victoriae - season | 2 | 0.2317 | 0.8 |  |
| Model: *H. steineni* season | 2 | 0.628 | 0.5654 |  |

**Table S11 - Statistics for alpha diversity results from warming experiment microbiome.**

| **Warming microbiome – Observed ASVs** | | | | |
| --- | --- | --- | --- | --- |
| Student’s t-test | Comparison | d.f. | t | *p* value |
|  | *C. georgiana* warming vs control | 4 | -4.502 | 0.0108 |
|  | *E. charcoti* warming vs control | 4 | 1.591 | 0.187 |
|  | *H. steineni* warming vs control | 4 | -0.402 | 0.708 |
| **Warming microbiome – Observed ASVs** | | | | |
| Student’s t-test | Comparison | d.f. | t | *p* value |
|  | *C. georgiana* warming vs control | 4 | 0.614 | 0.573 |
|  | *E. charcoti* warming vs control | 4 | 0.0328 | 0.975 |
| Mann-Whitney U  Rank Sum Test | Comparison | Mann- Whitney U Statistic | T | *p* (exact) |
|  | *H. steineni* warming vs control | 3 | 12.000 | 0.700 |

**Table S12 - Statistics for beta diversity results from warming experiment microbiome.**

| **Warming microbiome - Bray Curtis dissimilarity distances** | | | | |
| --- | --- | --- | --- | --- |
| PERMANOVA | d.f. | *r*^2^ | F | Pr(>F) |
| Model: species | 2 | 0.188781 | 1.745344 | 0.001 |
| Model: treatment | 1 | 0.070225 | 1.20847 | 0.132 |
| Model: species and treatment | 5 | 0.351292037 | 1.299661689 | 0.009 |
| Model: *C. georgiana* - treatment | 1 | 0.211541 | 1.073185 | 0.4 |
| Model: *E. charcoti* - treatment | 1 | 0.182935 | 0.89557 | 0.9 |
| Model: *H. steineni* - treatment | 1 | 0.21093 | 1.069258 | 0.3 |
| **Homogeneity of group dispersions in Bray Curtis dissimilarity distances** | | | | |
| One-Way ANOVA | d.f. | F | Pr(>F) |  |
| Model: species | 2 | 1.2779 | 0.3073 |  |
| Model: treatment | 1 | 0.8036 | 0.3833 |  |
| Model: *C. georgiana* - treatment | 1 | 0.069 | 0.8057 |  |
| Model: *E. charcoti* - treatment | 1 | 0.346 | 0.588 |  |
| Model: *H. steineni* - treatment | 1 | 1.5246 | 0.2845 |  |

**Table S13: List of ASVs shared between echinoderms during winter, season, and warming microbiome experiments.** ASVs were agglomerated to genus level and ASVs not identified at family or genus level are not shown.

| **Winter microbiomes** | | | | |
| --- | --- | --- | --- | --- |
| **ASVs shared between suspension feeders (*n* = 1)** | | | | |
| **Phylum** | **Class** | **Order** | **Family** | **Genus** |
| Actinomycetota | Actinobacteria | Micrococcales | Intrasporangiaceae | *Knoellia* |
| **ASVs shared between secondary consumers (n = 8)** | | | | |
| Bacillota | Clostridia | Clostridiales | Clostridiaceae | *Clostridium* |
| Fusobacteriota | Fusobacteriia | Fusobacteriales | Fusobacteriaceae | *Psychrilyobacter* |
| Planctomycetota | Planctomycetes | Pirellulales | Pirellulaceae | *Bythopirellula* |
| Actinomycetota | Acidimicrobiia | Microtrichales | Ilumatobacteraceae | *Ilumatobacter* |
| Planctomycetota | Planctomycetes | Pirellulales | Pirellulaceae | *Rubripirellula* |
| Pseudomonadota | Alphaproteobacteria | Rhodobacterales | Paracoccaceae | *Roseobacter* |
| Pseudomonadota | Alphaproteobacteria | Hyphomicrobiales | Methyloligellaceae | *Methyloceanibacter* |
| Bacteroidota | Bacteroidia | Flavobacteriales | Flavobacteriaceae | *Flavimarina* |
| **Seasonal microbiomes** | | | | |
| **ASVs shared between *O. validus*, *O. victoriae* and *H. steineni* across late summer, winter and early summer (*n* = 21)** | | | | |
| **Phylum** | **Class** | **Order** | **Family** | ***Genus*** |
| Pseudomonadota | Alphaproteobacteria | Rhodobacterales | Paracoccaceae | *Sulfitobacter* |
| Actinomycetota | Acidimicrobiia | Microtrichales | Ilumatobacteraceae | *Ilumatobacter* |
| Bacillota | Clostridia | Clostridiales | Clostridiaceae | *Clostridium* |
| Pseudomonadota | Alphaproteobacteria | Caulobacterales | Caulobacteraceae | *Caulobacter* |
| Fusobacteriota | Fusobacteriia | Fusobacteriales | Fusobacteriaceae | *Psychrilyobacter* |
| Actinomycetota | Actinobacteria | Mycobacteriales | Corynebacteriaceae | *Corynebacterium* |
| Planctomycetota | Planctomycetes | Pirellulales | Pirellulaceae | *Mariniblastus* |
| Verrucomicrobiota | Verrucomicrobiia | Verrucomicrobiales | Rubritaleaceae | *Rubritalea* |
| Planctomycetota | Planctomycetes | Pirellulales | Pirellulaceae | *Bythopirellula* |
| Pseudomonadota | Alphaproteobacteria | Hyphomicrobiales | Rhizobiaceae | *Ahrensia* |
| Planctomycetota | Planctomycetes | Pirellulales | Pirellulaceae | *Rubripirellula* |
| Actinomycetota | Actinobacteria | Propionibacteriales | Propionibacteriaceae | *Cutibacterium* |
| Campylobacterota | Campylobacteria | Campylobacterales | Arcobacteraceae | *Arcobacter* |
| Planctomycetota | Planctomycetes | Pirellulales | Pirellulaceae | *Pir4 lineage* |
| Pseudomonadota | Alphaproteobacteria | Rhodobacterales | Paracoccaceae | *Roseobacter* |
| Pseudomonadota | Alphaproteobacteria | Hyphomicrobiales | Methyloligellaceae | *Methyloceanibacter* |
| Pseudomonadota | Alphaproteobacteria | Rhodobacterales | Paracoccaceae | *Amaricoccus* |
| Planctomycetota | Planctomycetes | Pirellulales | Pirellulaceae | *Rubripirellula* |
| Pseudomonadota | Alphaproteobacteria | Rhodobacterales | Paracoccaceae | *Jannaschia* |
| **Warming experiment microbiomes** | | | | |
| **ASVs shared between *C. georgiana, E. charcoti* and *H. steineni* during the warming experiment (*n* = 45)** | | | | |
| **Phylum** | **Class** | **Order** | **Family** | *Genus* |
| Planctomycetota | Planctomycetes | Planctomycetales | Rubinisphaeraceae | *Rubinisphaera* |
| Planctomycetota | Planctomycetes | Pirellulales | Pirellulaceae | *Bythopirellula* |
| Pseudomonadota | Alphaproteobacteria | Rhodobacterales | Paracoccaceae | *Sulfitobacter* |
| Actinomycetota | Acidimicrobiia | Microtrichales | Ilumatobacteraceae | *Ilumatobacter* |
| Bacteroidota | Bacteroidia | Flavobacteriales | Flavobacteriaceae | *Patiriisocius* |
| Bacteroidota | Bacteroidia | Flavobacteriales | Flavobacteriaceae | *Polaribacter* |
| Planctomycetota | Planctomycetes | Planctomycetales | Gimesiaceae | *Gimesia* |
| Planctomycetota | Planctomycetes | Pirellulales | Pirellulaceae | *Rhodopirellula* |
| Bacteroidota | Bacteroidia | Flavobacteriales | Flavobacteriaceae | *Aureibaculum* |
| Pseudomonadota | Alphaproteobacteria | Rhodobacterales | Paracoccaceae | *Amaricoccus* |
| Planctomycetota | Planctomycetes | Planctomycetales | Rubinisphaeraceae | *Planctomicrobium* |
| Actinomycetota | Actinobacteria | Mycobacteriales | Nocardiaceae | *Rhodococcus* |
| Planctomycetota | Planctomycetes | Pirellulales | Pirellulaceae | *Mariniblastus* |
| Pseudomonadota | Alphaproteobacteria | Rhodobacterales | Paracoccaceae | *Pelagicola* |
| Planctomycetota | Planctomycetes | Planctomycetales | Rubinisphaeraceae | *Planctomicrobium* |
| Verrucomicrobiota | Verrucomicrobiia | Verrucomicrobiales | Rubritaleaceae | *Rubritalea* |
| Pseudomonadota | Alphaproteobacteria | Hyphomicrobiales | Pleomorphomonadaceae | *Pleomorphomonas* |
| Pseudomonadota | Gammaproteobacteria | Rickettsiellales | Rickettsiellaceae | *Aquicella* |
| Planctomycetota | Planctomycetes | Pirellulales | Pirellulaceae | *Rhodopirellula* |
| Planctomycetota | Planctomycetes | Pirellulales | Pirellulaceae | *Pir4 lineage* |
| Pseudomonadota | Alphaproteobacteria | Rhodobacterales | Paracoccaceae | *Roseobacter* |
| Planctomycetota | Planctomycetes | Planctomycetales | Rubinisphaeraceae | *Planctomicrobium* |
| Verrucomicrobiota | Verrucomicrobiia | Verrucomicrobiales | Rubritaleaceae | *Rubritalea* |
| Actinomycetota | Actinobacteria | Propionibacteriales | Nocardioidaceae | *Nocardioides* |
| Actinomycetota | Acidimicrobiia | Microtrichales | Microtrichaceae | Sva0996 marine group |
| Pseudomonadota | Alphaproteobacteria | Rhodobacterales | Paracoccaceae | *Amaricoccus* |
| Planctomycetota | Planctomycetes | Pirellulales | Pirellulaceae | *Rubripirellula* |
| Planctomycetota | Planctomycetes | Pirellulales | Pirellulaceae | *Mariniblastus* |
| Pseudomonadota | Alphaproteobacteria | Hyphomicrobiales | Methyloligellaceae | *Methyloceanibacter* |
| Planctomycetota | Planctomycetes | Planctomycetales | Rubinisphaeraceae | *Fuerstia* |
| Pseudomonadota | Alphaproteobacteria | Hyphomicrobiales | Hyphomicrobiaceae | *Filomicrobium* |
| Pseudomonadota | Alphaproteobacteria | Rhodobacterales | Paracoccaceae | *Tateyamaria* |
| Pseudomonadota | Alphaproteobacteria | Rhodobacterales | Paracoccaceae | *Parasedimentitalea* |

**Table S14 - Identities of cultured representatives from Hwengwere *et al.* (2025), with 100% 16S rRNA gene sequence similarity to ASV sequences identified in *C. georgiana*, *E. charcoti*, and *H. steineni* in amplicon sequence data.** 10 cultured representatives shared 100% gene sequence similarity to 11 ASVs from the warming experiment study.

| **Identifier** | **NCBI Accession num.** | **Class** | **Genus** |
| --- | --- | --- | --- |
| 1S14 | PQ101178 | *Actinobacteria* | *Alpinimonas* |
| 4S16 | PQ101189 | *Actinobacteria* | NA |
| 4S19 | PQ101190 | *Actinobacteria* | *Pseudarthrobacter* |
| 5G4 | PQ101195 | *Actinobacteria* | *Alpinimonas* |
| 6F6 | PQ101206 | *Alphaproteobacteria* | *Sphingomonas* |
| 6G9 | PQ101209 | *Actinobacteria* | *Rhodoglobus* |
| NG4 | PQ101230 | *Planctomycetes* | *Fuerstia* |
| NG4 | PQ101230 | *Actinobacteria* | *Rhodococcus* |
| SG15 | PQ101239 | *Actinobacteria* | *Pseudarthrobacter* |
| SG16 | PQ101240 | *Actinobacteria* | *Salinibacterium* |
| SG16 | PQ101240 | *Actinobacteria* | *Rhodococcus* |

**Supplementary Figures**

**
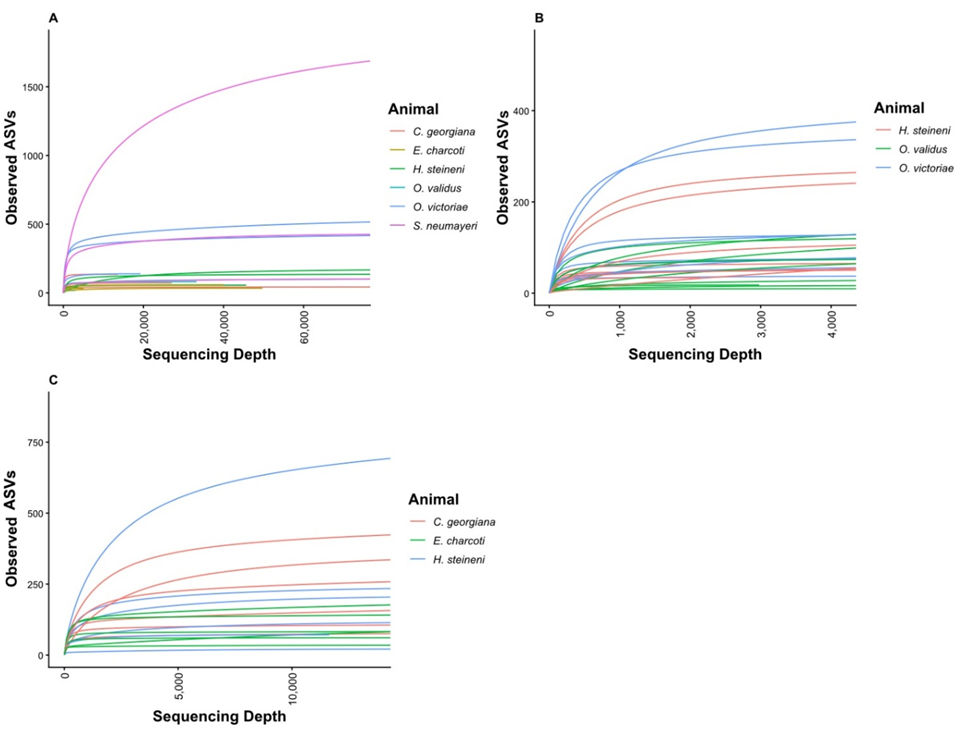
**

**Figure S1 - Rarefaction curves of the 54 sequenced samples prior to normalisation.** Observed ASVs at increasing sequencing depth in **(A)** winter microbiome **(B)** seasonal microbiome and **(C)** warming experiment microbiome are shown.

**
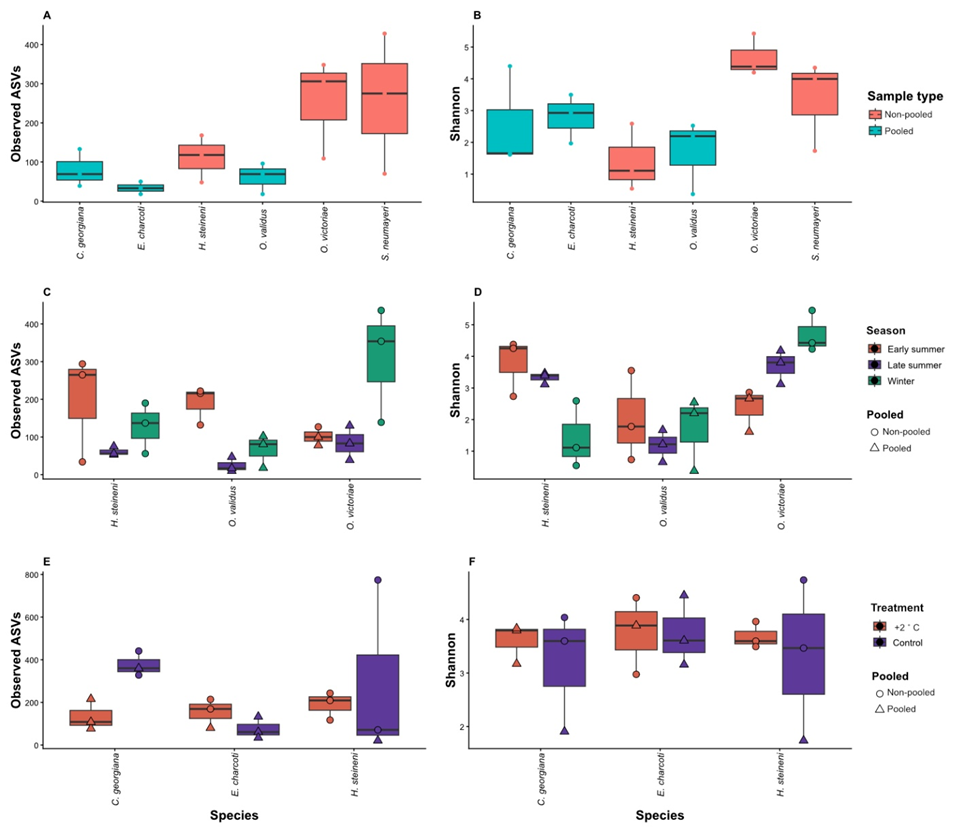
**

**Figure S2 - Effects of pooling on alpha diversity metrics Observed AVS and Shannon diversity.** **(A-B)** Winter microbiome experiment, **(C-D)** Season microbiome, and **(E-F)** warming experiment microbiome Observed ASVs and Shannon diversity after CSS normalisation.

**
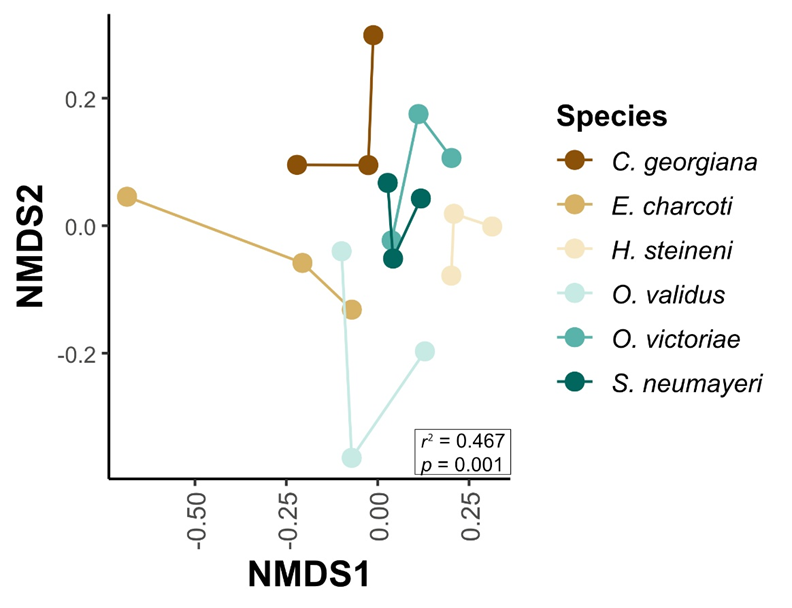
**

**Figure S3: Beta-diversity metrics of C*. georgiana*, *E. charcoti*, *H. steineni*, *O. validus*, *O. victoriae*, and *S. neumayeri* in winter.** NMDs plot of beta diversity analysis using Bray-Curtis dissimilarity distances. Across the six echinoderms, PERMANOVA analysis showed statistical significance between species (*p* value indicated within NMDS plot). For full details of statistical analysis results see Supplementary Data, Table S7.


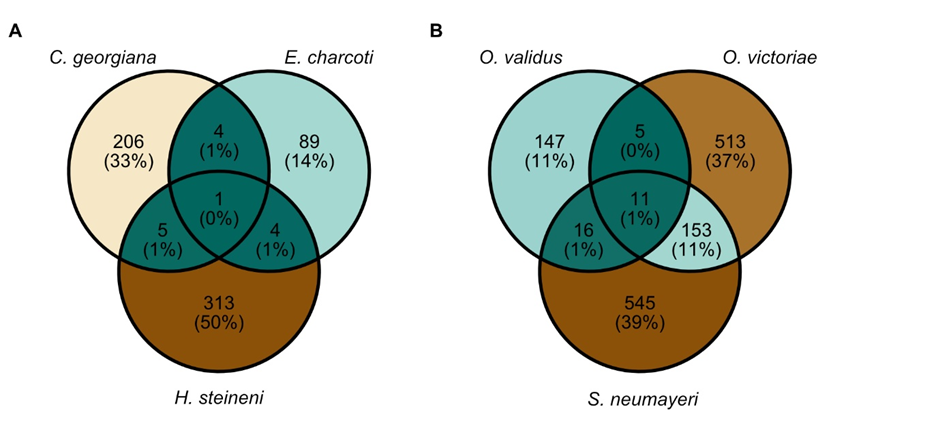


**Figure S4: Shared ASVs between suspension feeders and omnivores and predator/scavenger. (A)** Shared ASVs between three sea cucumbers, *C. georgiana*, *E. charcoti*, and *H. steineni*. **(B)** Shared ASVs between the omnivores (*O. victoriae* and *S. neumayeri*) and the scavenger/predator (*O. validus*). The number of ASVs and their percentage of the total ASVs identified are provided.


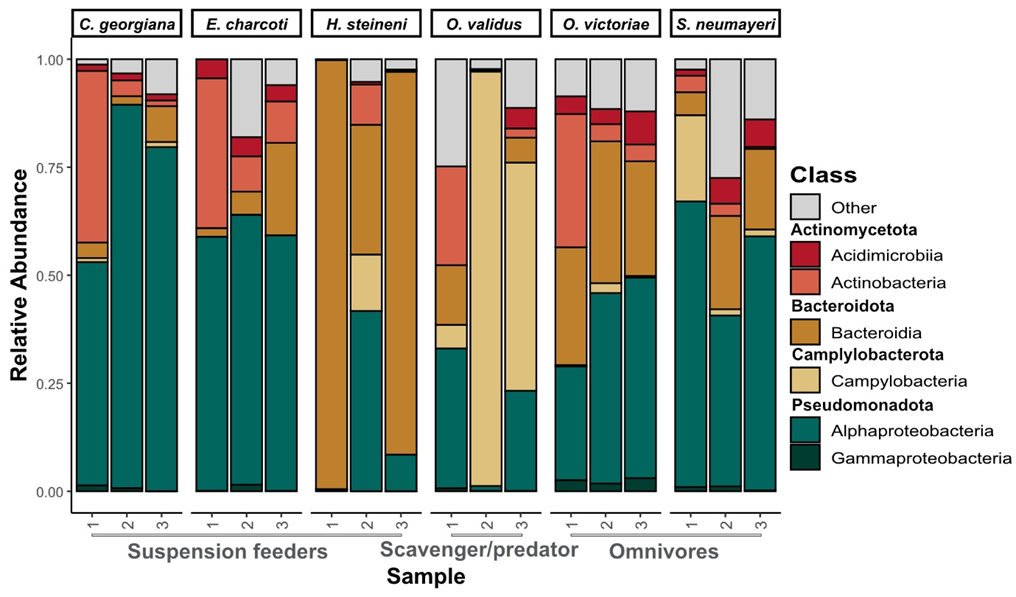


**Figure S5:** The relative abundances of bacteria are provided at class level (with corresponding bacterial phyla indicated) based on 16S rRNA gene V3-V4 region sequence identities. Only bacterial classes that presented median relative abundance values greater than 1% within each species are shown, whilst those that fell below this criterion were grouped into ‘Other’.


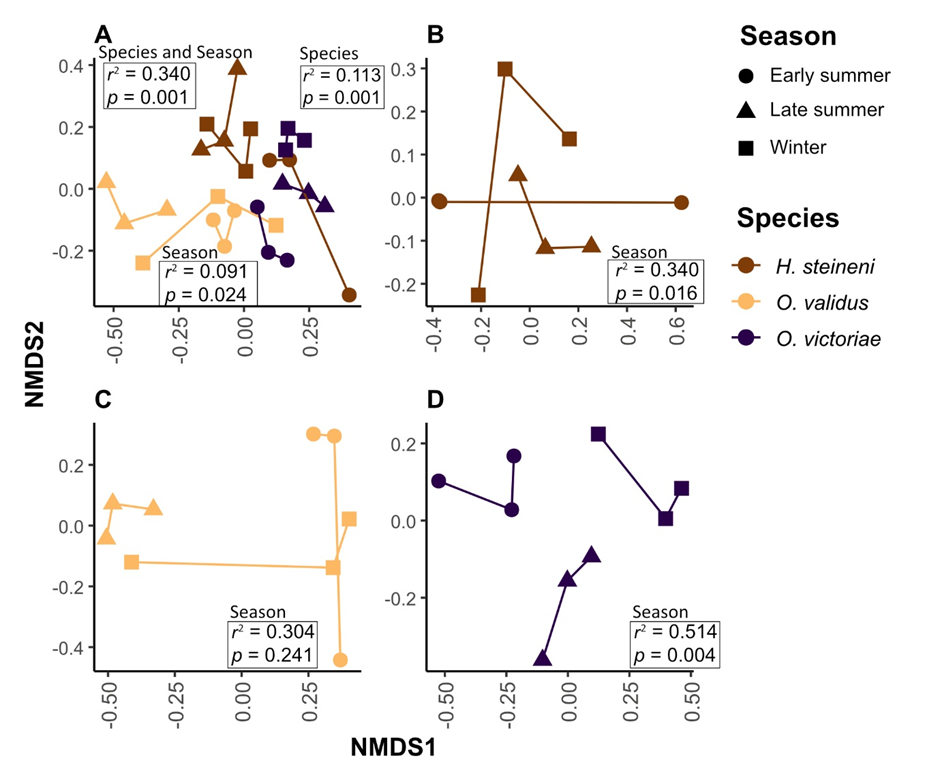


**Figure S6: Beta-diversity metrics of *H. steineni*, *O. validus*, and *O. victoriae* in late summer, winter, and early summer. (A)** Across the three species and seasons, PERMANOVA analysis showed statistical significance between species (*p* value indicated within NMDS plot). **(B-D)** Further separation of samples according to species showed statistically significant differences according to season for *H. steineni* and *O. victoriae* (PERMANOVA, P<0.05). Interactions that were not statistically significant are not displayed in the figures. Full details of statistical analysis results are provided in Supplementary Data Table S9.


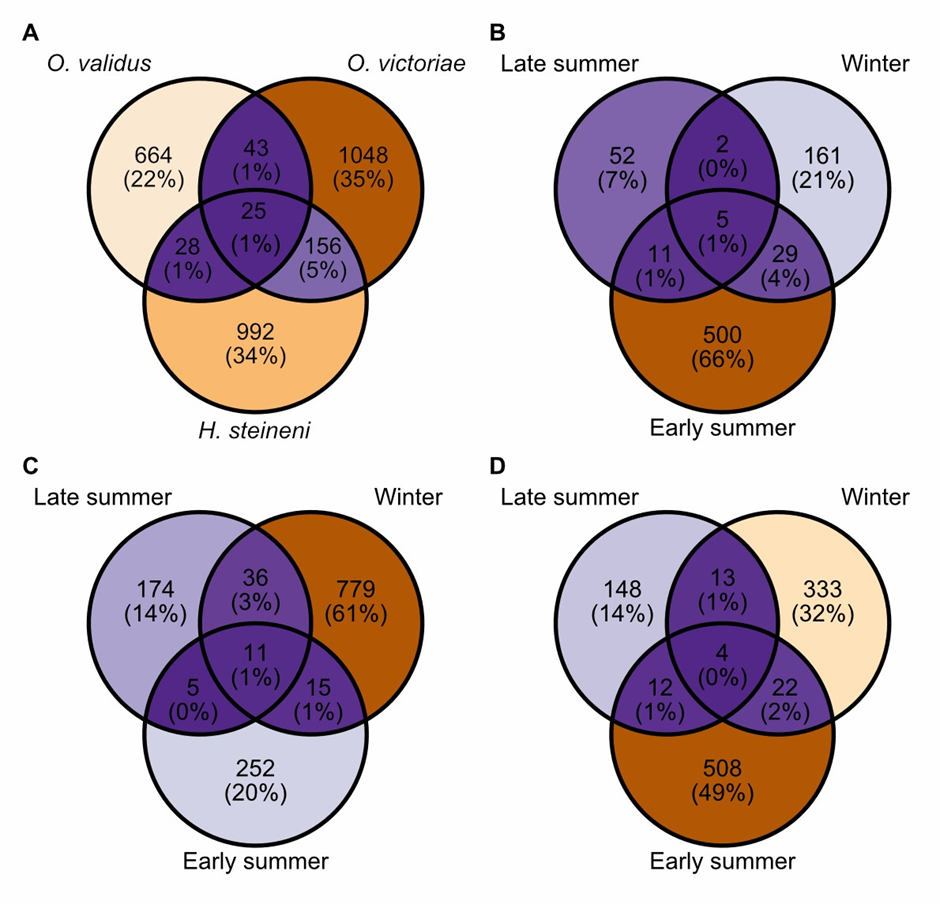


**Figure S7: Shared ASVs between late summer, winter and early summer are provided for (B) *O. validus*, (C) *O. victoriae*, (D) and *H. steineni*.** The number of ASVs and their percentage of the total ASVs identified are provided.

**
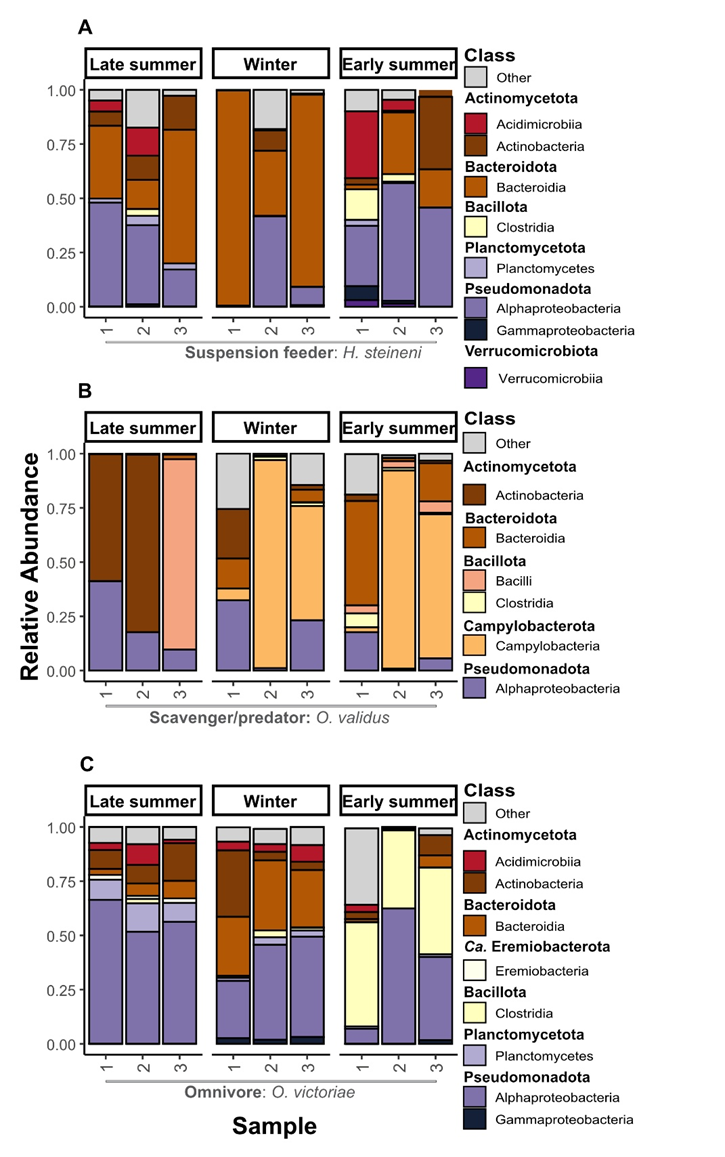
**

**Figure S8: Taxonomic composition of (A) *H. steineni* (B) *O. validus*, and (C) *O. victoriae* gut microbiomes in late summer, winter, and early summer.** The relative abundances of bacteria are provided at class level (with corresponding bacterial phyla indicated) based on 16S rRNA gene V3-V4 region sequence identities. Only bacterial classes that presented median relative abundance values greater than 1% within each species are shown, whilst those that fell below this criterion were grouped into ‘Other’. Please note the *Candidatus* phylum *Eremiobacterota* is now reclassified as phylum *Vulcanimicrobiota,* and the class *Eremiobacteria* has now been reclassified to *Vulcanimicrobiia*.


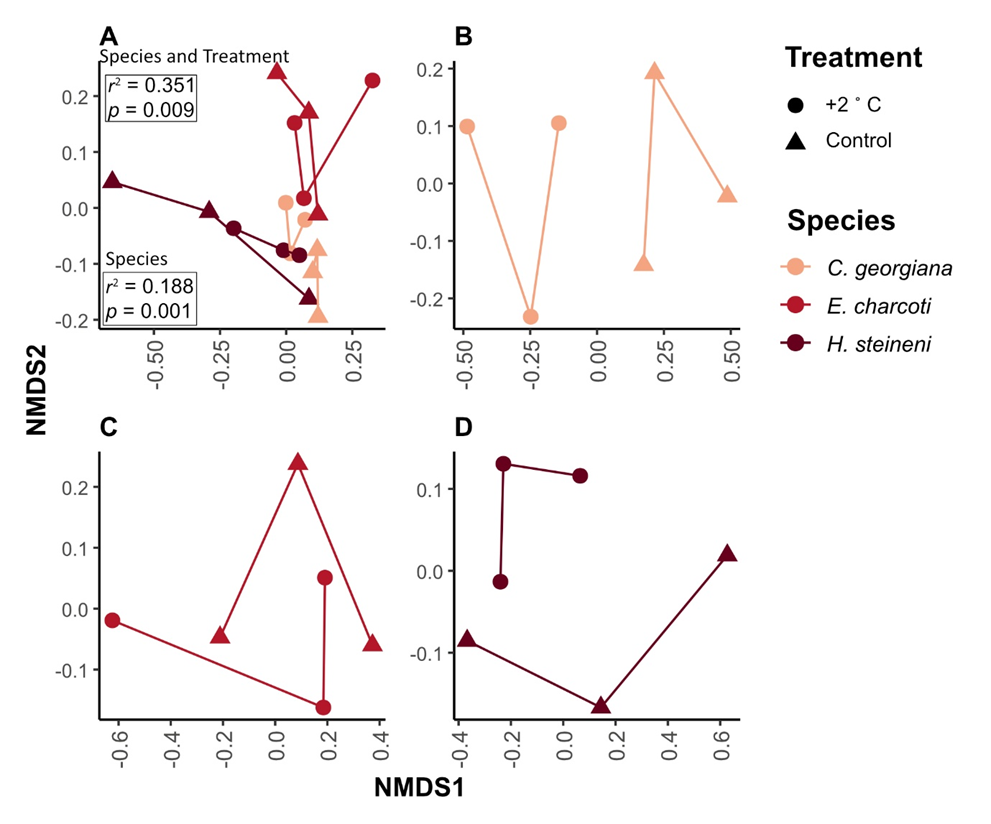


**Figure S9: Beta-diversity metrics of *C. georgiana, E. charcoti, and H. steineni* following a six-month warming experiment. (A)** Across the three species and seasons, PERMANOVA analysis showed statistical significance between species (*p* value indicated within NMDS plot). **(B-D)** Further separation of samples according to species showed no statistically significant differences according to treatment for all three sea cucumber species which were analysed together in **(A)**. Interactions that were not statistically significant are not displayed in the figures. Full details of statistical analysis results are provided in Supplementary Data Table S12.


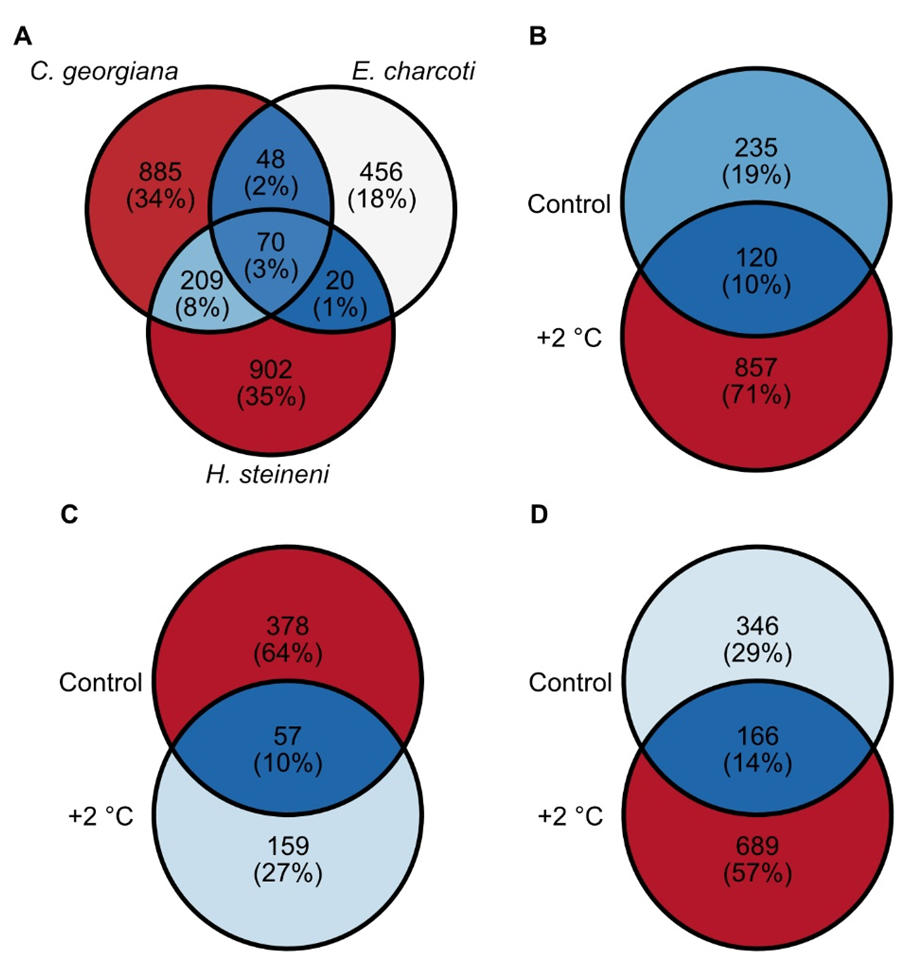


**Figure S10: Shared ASVs between sea cucumbers in warming microbiome experiment. (A)** Shared ASVs between all three sea cucumbers, *C. georgiana*, *E. charcoti* and *H. steineni*, in warming and control conditions. **(B)** Shared ASVs between *C. georgiana* in warming and control conditions. **(C)** Shared ASVs between *E. charcoti* in warming and control conditions. **(D)** Shared ASVs between *H. steineni* in warming and control conditions. The number of ASVs and their percentage of the total ASVs identified are provided.


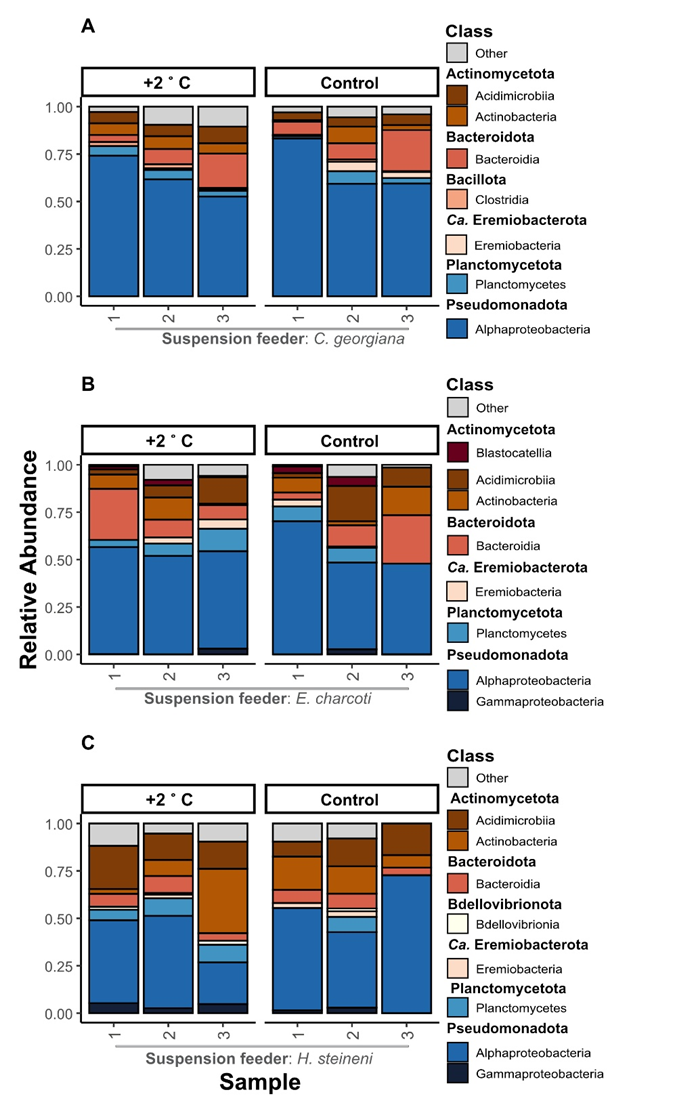


**Figure S11:Taxonomic composition of *C. georgiana*, *E. charcoti* and *H. steineni*, following a six-month warming experiment**. **in late summer, winter, and early summer.** The relative abundances of bacteria in **(A)** *C.georgiana* **(B)** *E. charcoti*, and **(C)** *H. steineni* gut microbiomes bacteria are provided at class level (with corresponding bacterial phyla indicated) based on 16S rRNA gene V3-V4 region sequence identities. Only bacterial classes that presented median relative abundance values greater than 1% within each species are shown, whilst those that fell below this criterion were grouped into ‘Other’. Please note the *Candidatus* phylum *Eremiobacterota* is now reclassified as phylum *Vulcanimicrobiota*, and the class *Eremiobacteria* has now been reclassified to *Vulcanimicrobiia*.

**Figure S12 - Upper thermal limits (UTLs) of six Antarctic echinoderms with gradually slowing rates of warming.**

This figure shows the mean UTLs of three sea cucumbers, *C. georgiana*, *E. charcoti*, and *H. steineni* at three rates of warming 1 ˚ C h^-1^, 1 ˚ C Day^-1^, and 1 ˚ C 3 Days^-1^ which were determined according to previously published protocols (Peck et al., 2013). The error bars represent the standard error of the mean. Sample numbers for these rates of warming were as follows, *C. georgiana* (20, 20, 19), *E. charcoti* (27, 20, 20), and *H. steineni* (26, 20, 20). All three sea cucumber species decreased in thermal resilience as the rate of warming slowed down. *H. steineni* and *C. georgiana* were most tolerant to the slowest rates of warming (1 ˚ C 3 Days^-1^), with *E. charcoti* being an average of ~3 ˚C less thermally tolerant.


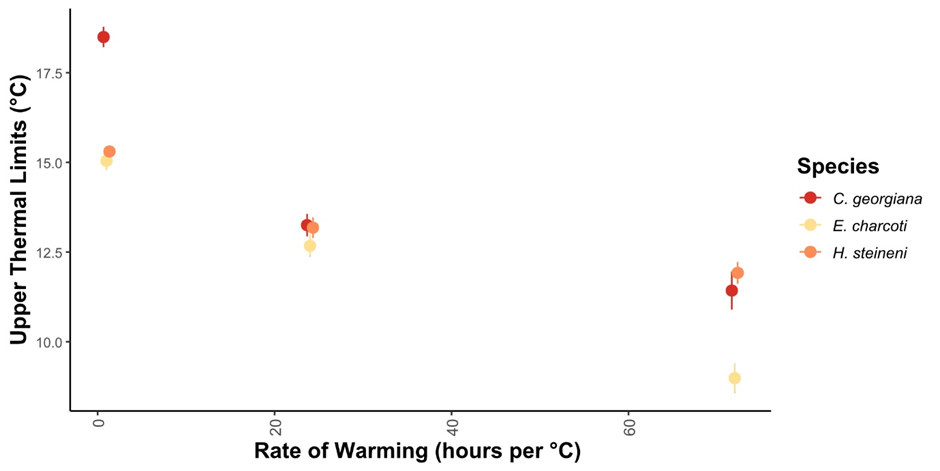

Supplement: Supplementary file 1 — Supplementary Material 1. [file 12866_2026_5114_MOESM1_ESM.docx]
